# Supplementary material for: Heterogeneous, temporally consistent, and plastic brain development after preterm birth
Source: Nat Commun. 2025 Sep 12;16:8269. doi: 10.1038/s41467-025-63967-1 (PMC12432136; doi:10.1038/s41467-025-63967-1)
Supplement: Supplementary file 1 — Supplementary Information [file 41467_2025_63967_MOESM1_ESM.pdf]

Supplementary Materials

Heterogeneous, temporally consistent, and plastic brain development after preterm birth

Melissa Thalhammer, MSc*; Jakob Seidlitz, PhD; Antonia Neubauer, MD; Aurore Menegaux, PhD; Benita Schmitz-Koep, MD; Maria A. Di Biase, PhD; Julia Schulz, MSc; Lena Dorfschmidt, PhD; Richard A. I. Bethlehem, PhD; Aaron Alexander-Bloch, PhD; Chris Adamson, PhD; Gareth Ball, PhD; Joana A. Sa de Almeida, PhD; Richard Beare, PhD; Claus Zimmer, MD; Marcel Daamen, Dr. phil.; Henning Boecker, MD; Peter Bartmann, MD, PhD; Dieter Wolke, PhD; Dennis M. Hedderich, MD; Christian Sorg, MD

*** Corresponding author:**

melissa.thalhammer@tum.de, melissa.a.thalhammer@gmail.com

Table of contents

1. Abbreviations ... 5

2. Supplementary Methods and Results ... 6

2.1. Heterogeneity of cortical aberrations after preterm birth when excluding cases of severe perinatal brain injury ... 6

2.2. Deviation score estimation using different normative reference charts by Rutherford et al. for BLS-26 ... 7

2.3. Heterogeneity of regions across preterm subjects ... 8

2.4. Spatial association between adult CTh IBAPs and cell type-specific gene expression using an alternative spatial null model ... 8

2.5. Software ... 9

3. Supplementary Figures ... 10

Fig. S1: Individual heterogeneity of cortical thickness and surface area in subjects without neonatal brain injury ... 10

Fig. S2: Extranormal individual deviations estimated with a different population reference normative model ... 12

Fig. S3: Correlation of cortical thickness centile estimates between the Bethlehem-framework and the Rutherford-framework ... 14

Fig. S4: Correlation of surface area centile estimates between the Bethlehem-framework and the Rutherford-framework ... 16

Fig. S5: Spatial heterogeneity of cortical thickness individual brain abnormality patterns between preterm subjects ... 18

Fig. S6: Spatial heterogeneity of surface area individual brain abnormality patterns between preterm subjects ... 19

Fig. S7: Similarity of inter-regional deviation patterns of cortical thickness ... 20

Fig. S8: Similarity of inter-regional deviation patterns of surface area ... 21

Fig. S9: Individual heterogeneity of cerebral tissue volume measures after preterm birth ... 22

Fig. S10: Relationship between cortical thickness individual brain abnormality patterns and gestational age ... 23

Fig. S11: Relationship between cortical thickness individual brain abnormality patterns and gestational age in different stages of prematurity ... 24

Fig. S12: Anatomical lesion consistency of individual deviations in cortical thickness from 10 to 12 years 26

Fig. S13: Anatomical lesion consistency of individual deviations in cortical thickness from 26 to 38 years 27

Fig. S14: Anatomical lesion consistency of individual deviations in surface area from 10 to 12 years 28

Fig. S15: Anatomical lesion consistency of individual deviations in surface area from 26 to 38 years 29

Fig. S16 Regional distribution of Intraclass Correlation Coefficients for longitudinal cortical thickness deviations 30

Fig. S17: Significant associations between individual cortical thickness deviations of preterm adults and mean expression profile of eight brain cell types 31

Fig. S18: Principal component loadings 32

Fig. S19: Control analyses for the associations between the first principal component across regional cortical thickness and socio-economic status 33

Fig. S20: Control analyses for the associations between the first principal component across regional cortical thickness and IQ 34

4. Supplementary Tables 35

Table S1: Regions of the Desikan-Killiany atlas[25] 35

Table S2: Statistics for average dysmaturation outcomes 36

Table S2a: Average dysmaturation outcomes of cortical thickness in the dHCP cohort 36

Table S2b: Average dysmaturation outcomes of surface area in the dHCP cohort 38

Table S2c: Average dysmaturation outcomes of cerebral tissue volume measures in the dHCP cohort 39

Table S2d: Average dysmaturation outcomes of cortical thickness in the ABCD-10 cohort 40

Table S2e: Average dysmaturation outcomes of surface area in the ABCD-10 cohort 41

Table S2f: Average dysmaturation outcomes of cerebral tissue volume measures in the ABCD-10 cohort 42

Table S2g: Average dysmaturation outcomes of cortical thickness in the ABCD-12 cohort 43

Table S2h: Average dysmaturation outcomes of surface area in the ABCD-12 cohort 44

Table S2i: Average dysmaturation outcomes of cerebral tissue volume measures in the ABCD-12 cohort......45

Table S2j: Average dysmaturation outcomes of cortical thickness in the BLS-26 cohort......46

Table S2k: Average dysmaturation outcomes of surface area in the BLS-26 cohort......47

Table S2l: Average dysmaturation outcomes of cerebral tissue volume measures in the BLS-26 cohort......48

Table S2m: Average dysmaturation outcomes of cortical thickness in the BLS-38 cohort......49

Table S2n: Average dysmaturation outcomes of surface area in the BLS-38 cohort......50

Table S2o: Average dysmaturation outcomes of cerebral tissue volume measures in the BLS-38 cohort......51

Tables S3: Comparison between the Bethlehem-framework and the Rutherford-framework......52

Table S3a: Spearman correlation coefficients between cortical thickness deviation score estimates based on the Bethlehem-framework and the Rutherford-framework......52

Table S3b: Spearman correlation coefficients between surface area deviation score estimates based on the Bethlehem-framework and the Rutherford-framework......54

Supplementary Tables S4: Intraclass correlation coefficients (ICC) for longitudinal IBAP consistency within individuals......55

Table S4a: Intraclass correlation coefficients of surface area in the ABCD cohort......55

Table S4b: Intraclass correlation coefficients of surface area in the BLS cohort......57

Table S4c: Intraclass correlation coefficients of cortical thickness in the ABCD cohort......58

Table S4d: Intraclass correlation coefficients of cortical thickness in the BLS cohort......59

Supplementary Table S5: Association between strength of the spatial relationship between cellular distributions and cortical thickness deviation profiles with gestational age......60

5. Supplementary References......61

1. Abbreviations

ABCD	Adolescent Brain Cognitive Development Study
AHBA	Allen Human Brain Atlas
Astro	Astrocytes
BLS	Bavarian Longitudinal Study
BW	Birth weight
CI	Confidence interval
CTh	Cortical thickness
CTV	Cerebral tissue volume
dHCP	Developing Human Connectome Project
DNTI	Duration of neonatological treatment index
Endo	Endothelial cells
FDR	False discovery rate
FT	full-term
g	Grams
GA	Gestational age
GMV	Grey matter volume
IBAPs	Individual brain abnormality patterns
Micro	Microglia
Neuro-Ex	Excitatory neurons
Neuro-In	Inhibitory neurons
Oligo	Oligodendrocytes
OPC	Oligodendrocyte progenitor cells
PC1	Principal component 1
PCA	Principal Component Analysis
Per	Pericytes
PIRI	Parent-infant relationship index
PT	preterm
ROIs	Regions of interest
SA	Surface area
SES	Socio-economic status
sGMV	Subcortical grey matter volume
VLBW	Very low body weight (< 1,500 g)
VP	Very preterm (< 32 weeks of gestation)
WMV	White matter volume

2. Supplementary Methods and Results

2.1. Heterogeneity of cortical aberrations after preterm birth when excluding cases of severe perinatal brain injury

Since perinatal brain injuries such as intracranial hemorrhage or focal white matter lesions, which are often caused by preterm birth, might impact brain morphometry and cognitive outcomes, we performed a control analysis of the main results excluding subjects with perinatal brain injuries.

For the dHCP cohort, the variable "radiology_score" was used, which indicates the presence of incidental findings that might be of clinical significance and/or might affect the image reconstruction. Radiology score was rated on a 1–5-point scale with the following meanings: (1) normal appearance for age, (2) incidental findings with unlikely significance for clinical outcome or analysis (e.g., subdural hemorrhage, isolated subependymal cysts, mild inferior vermis rotation), (3) incidental findings with unlikely clinical significance but possible analysis significance (e.g. several punctate lesions or other focal white matter/ cortical lesions not thought to be of clinical significance), (4) incidental findings with possible clinical significance, unlikely analysis significance (e.g. isolated non brain anomaly, e.g., in pituitary / on tongue), (5) incidental findings with possible/likely significance for both clinical and imaging analysis (e.g., major lesions within white / matter, cortex, cerebellum and or basal ganglia, small head/brain < 1st centile) (file "nnsi01_definitions.csv" downloaded from https://nda.nih.gov/edit_collection.html?id=3955). Subjects with a radiology score of > 2 were excluded for the control analysis ($n_{preterm}$ = 64, $n_{full\text{-}term}$ = 78). For the ABCD cohort, no information on perinatal brain injury was available. For the BLS cohort, since no MRI scan was performed at birth, information on neonatal brain injury was limited. Only a few preterm subjects had an intracranial hemorrhage (ICH) according to ultrasound examinations graded on a 1-4-point scale. As previous work by our group has shown, the presence but not the grading of ICH is a significant predictor of ventricular enlargement in adulthood[1], suggesting that the presence of neonatal brain injury impacts brain structure into adulthood. Therefore, in this control analysis, we excluded all subjects with neonatal ICH of any grading ($n_{preterm}$ = 15).

After exclusion of subjects with perinatal brain injury, analyses investigating average dysmaturation, interindividual heterogeneity, as well as correlation with cognitive outcomes were conducted as described in the main methods section.

Whereas average dysmaturation outcomes differed compared to the main analyses (see Fig. 2-4), individual heterogeneity results remained fairly stable, with no more than 27 % of preterm subjects sharing extranormal deviations in any given region (Supplementary Fig. S1). In preterm neonates, the associations of the first principal component of IBAPs across regions with cognitive outcome measures were not significant, similar to the original findings (see Fig. 9; CTh: Spearman rho(65) = -0.121, p = 0.392, p_{FDR} = 0.392, CI = [-0.351,0.123], two-tailed; SA: Spearman rho(65) = 0.141, p = 0.318, p_{FDR} = 0.392, CI = [-0.102,0.369], two-tailed). In preterm adults, the association between PC1

of SA IBAPs with full-scale IQ was still significant but only before FDR-correction (CTh: Spearman rho(79) = 0.183, p-value = 0.111, p_{FDR} = 0.222, CI = [-0.037,0.386], two-tailed; SA: Spearman rho(79) = 0.274, p = 0.016, p_{FDR} = 0.064, CI = [0.059,0.464], two-tailed).

2.2. Deviation score estimation using different normative reference charts by Rutherford et al. for BLS-26

Several research groups have addressed the need for well-defined reference models to quantify the variability of brain measurements across the lifespan[2–4]. To validate our results of individual brain abnormality pattern (IBAP) heterogeneity based on predictions from the BrainChart framework[3] with predictions from another popular, population-based, pretrained model, we deployed the braincharts framework by Rutherford and colleagues[2]. Due to the similar naming of the two frameworks, the BrainChart model by Bethlehem and colleagues[3] used for the main analysis will be termed "Bethlehem-framework", the braincharts model by Rutherford and colleagues[2] used for the control analysis will be termed "Rutherford-framework" in the following. Based on a large neuroimaging cohort of about 46,000 subjects from 59 sites, the Rutherford-framework also provides Desikan-Killiany-based regional CTh and SA estimates for the human lifespan from 2-100 years. Bayesian Linear Regression with likelihood warping was used to predict CTh and SA from a vector of covariates (sex, age, and site). Pretrained models were obtained from the developers (https://github.com/predictive-clinical-neuroscience/braincharts) and adapted to the BLS-26 dataset using full-term adults. Subsequently, predictions for both cohorts were obtained, resulting in Z-scores of individual CTh or SA deviations, respectively. Analogue to the main analysis, subjects were classified as (i) infranormal, i.e., < 5th percentile corresponding to Z < -1.645, (ii) supranormal, i.e., > 95th percentile corresponding to Z > 1.645, or (iii) normal for each cortical region. For each region, the number of extranormal deviations were calculated as described in the Methods section.

Percentages of significant deviations per unilateral cortical region for preterm adults are visualized in Supplementary Fig. S2. In accordance with the main analysis, not more than 27 % of preterm adults significantly deviate from the norm in regional CTh or SA for any one unilateral region, corroborating the notion of substantial heterogeneity among preterm subjects with respect to regional cortical thickness.

Next, to compare deviation score estimates between the two different frameworks, "Rutherford-framework" Z-scores of each Desikan-Killiany ROI were averaged across hemispheres and transformed into centile scores using the cumulative distribution function of the standard normal distribution. We computed the Spearman correlation coefficient between the "Bethlehem-framework" centile score estimate and the "Rutherford-framework" centile score estimate for each ROI across full-term and preterm subjects of the BLS-26 cohort (Supplementary Fig. S3 and S4). Centile score estimates from the two frameworks showed strong correlation across ROIs for both CTh and SA, with Spearman correlation coefficients ranging from 0.660 to 0.886 for SA and from 0.881 to 0.995 for CTh

(see Supplementary Tables S3). Notably, the largest differences between frameworks occurred within the normal centile range, while most individuals with extranormal scores were consistently identified as such in both frameworks, suggesting that the presented deviation scores are largely independent on the population reference normative model used for their estimation.

2.3. Heterogeneity of regions across preterm subjects

To examine the similarity of inter-regional deviation patterns of CTh and SA across individuals, we computed the average Spearman correlation coefficient for each brain region across the preterm population in each cohort. Specifically, we cross-correlated binarized IBAPs across subjects (see Methods for a detailed description on binarization). The resulting average correlation coefficient per brain region indicate that patterns of extranormal deviations were generally not shared among individuals, with an average Spearman correlation of $r < 0.3$ for most region pairs, both for CTh and SA (Supplementary Fig. S7 - S8). An exception can be observed in the dHCP cohort, where average coefficients reach up $r = 0.75$. This likely reflects that, although there is substantial heterogeneity among regions that show an extranormal deviation between individuals, certain brain regions may exhibit some covariation in their deviation patterns within the cohort. In general, this control analysis supports our main finding that there is no to little consistent inter-regional pattern of extranormal deviations across preterm individuals.

2.4. Spatial association between adult CTh IBAPs and cell type-specific gene expression using an alternative spatial null model

In the main analysis, the spin test was used to assess statistical significance of the spatial association between adult CTh IBAPs and cell type-specific gene expression. While widely used, we agree that this method has theoretical limitations. For example, the projection to the surface[5] and the rotation of the medial wall onto the cortex[6], in some cases leads to the disruption of spatial autocorrelation when generating spatial surrogates with the spin test. While imperfect, spatial null models like the spin test have been shown to better control for false-positive rates compared to parametric or non-parametric tests when assessing statistical significance of map-to-map correspondence[7,8].

As a control analysis, we provide the significance of correlations between adult CTh IBAPs and regional cell type-specific gene expression as assessed with an alternative spatial null model, namely Brain Surrogate Maps with Autocorrelated Spatial Heterogeneity (BrainSMASH) as implemented in the neuromaps toolbox[9]. The method generates surrogate brain maps with similar spatial autocorrelation than the original one via variogram estimation[7,10]. Similarly to the main results, the association of some subjects' CTh IBAPs with some regional profiles of cellular density were significantly similar (Supplementary Methods and Results, Supplementary Fig. S17).

2.5. Software

All analyses were conducted in a Python 3.11.9 environment unless stated otherwise. For harmonizing CTh and SA values across scanners, NeuroCombat (0.2.12, for BLS-26)[11] or longCombat (0.0.0.9 in R version 4.4.1, for ABCD)[12] were used. Gene expression information was retrieved and processed with abagen (0.1.3)[13]. To assess map-to-map correspondence between two brain maps, the spin test implemented in the ENIGMA toolbox (2.0.3)[14] or the alternative spatial null model BrainSMASH as implemented in the neuromaps toolbox[9] (0.0.5, Supplementary Methods S2.4) were used. For data processing and statistical analyses, several routines from nibabel (5.3.2, https://doi.org/10.5281/zenodo.591597), scipy (1.12.0)[15], statsmodels (0.14.4)[16], statsannotations (0.7.1, https://zenodo.org/badge/latestdoi/296015778), pingouin (0.5.4)[17], scikit-learn (1.5.2), numpy (1.23.5), pandas (1.5.3) were used. The PROCESS Macro (4.3)[18] for R (4.4.1) was used to conduct the moderation analysis. Visualizations were created using matplotlib (3.9.2)[19], seaborn (0.13.2)[20], and the ENIGMA toolbox (2.0.3)[21]. For Fig. 10, the Simple Brain Plot toolbox (https://zenodo.org/badge/latestdoi/377448069) was used in MATLAB 2023b. Normative models from the BrainChart project were re-fit in R (4.4.1) using gamlss (5.4-22)[22], tidyverse (2.0.0)[23], rcompanion (2.4.36), and parallel (4.4.1) packages. The PCNtoolkit (0.30.post2)[24] was used to retrieve and re-fit an alternative normative model for regional CTh and SA (see Supplementary Methods S2.2).

3. Supplementary Figures

Fig. S1: Individual heterogeneity of cortical thickness and surface area in subjects without neonatal brain injury

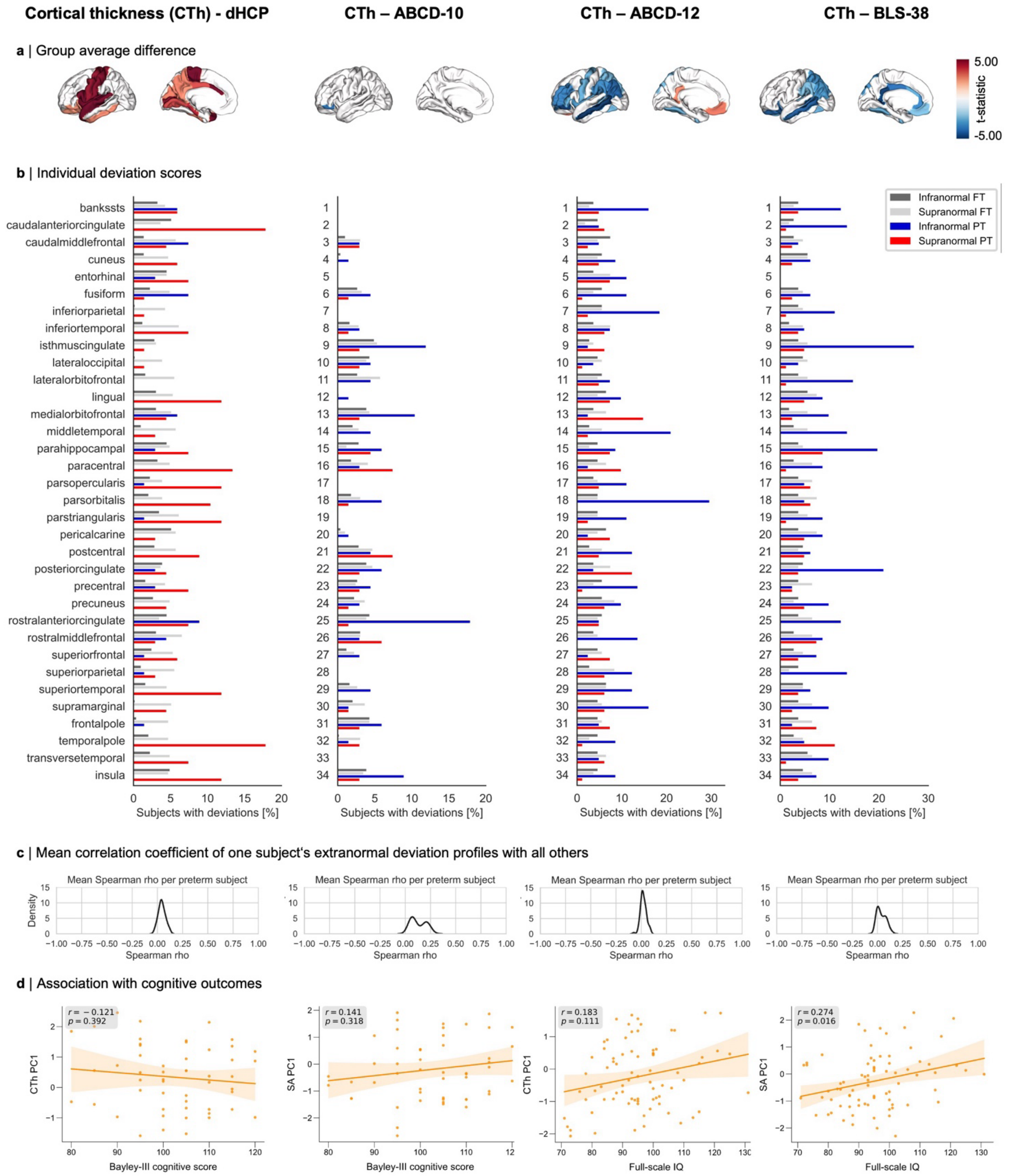

Supplementary Figure S1: Individual heterogeneity of cortical thickness and surface area in subjects without neonatal brain injury. Subjects with severe neonatal brain injury were excluded from this control analysis (see Supplementary Methods and Results S2.1 for detailed exclusion criteria). **a,** Average dysmaturation outcomes for cortical

thickness (CTh) and surface area (SA) after preterm birth estimated by linear regression models correcting for age and sex ($p_{FDR} < 0.05$, two-sided). **b,** Percentage of subjects with an infranormal (i.e., < 5th percentile; blue: preterm, dark gray: full-term) or supranormal (i.e., > 95th percentile; red: preterm, light gray: full-term) deviation in any given region are shown for preterm (PT) and full-term (FT) individuals. Similar to the main analysis, not more than 30 % of preterm subjects without severe neonatal brain injury share significant deviations in any given region. **c,** Distribution of averaged Spearman correlation coefficients of binarized extranormal deviation profiles for each subject with all others. **d,** Spearman correlation between measures of cognitive outcomes and the first principal component (PC1) of cortical individual brain abnormality patterns (IBAPs). Results are shown for neonates (dHCP, $n_{preterm} = 67$, $n_{full\text{-}term} = 486$) and adults (BLS-26, $n_{preterm} = 81$, $n_{full\text{-}term} = 107$). See Supplementary Table S1 for region abbreviations. Source data and exact p-values are provided as a Source Data file.

Fig. S2: Extranormal individual deviations estimated with a different population reference normative model

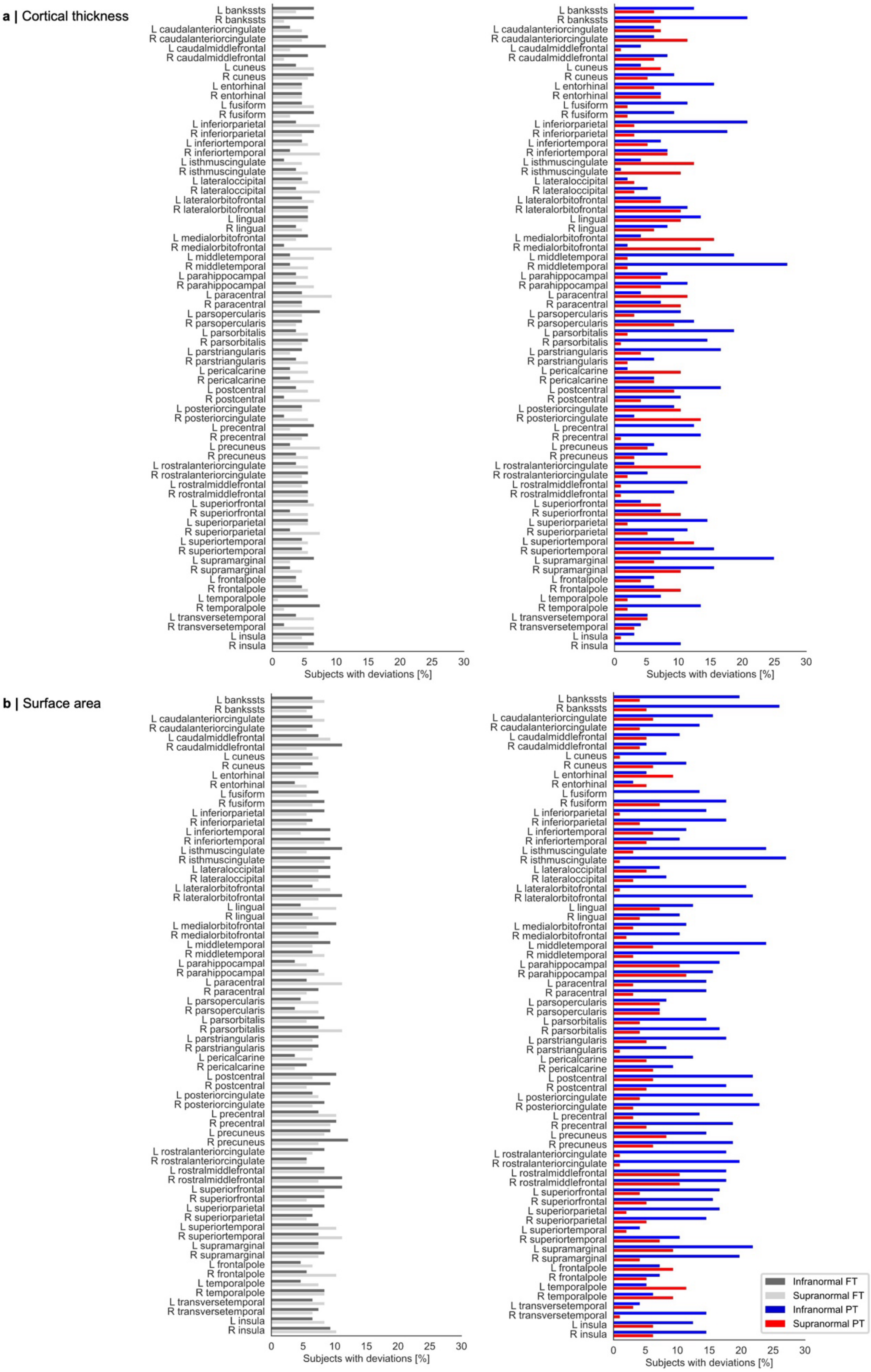

Supplementary Figure S2: Extranormal individual deviations estimated with a different population reference normative model. As a control analysis, a different population normative model[2] was used to estimate individual deviations for each preterm (PT) and full-term (FT) subject of the BLS-26 cohort for (**a**) cortical thickness and (**b**) surface area. As this normative model was trained on hemispheric data (L left, R right), data is shown for both hemispheres separately. Bars represent the percentage of subjects with an infranormal (i.e., < 5th percentile; blue: preterm, dark gray: full-term) or supranormal (i.e., > 95th percentile; red: preterm, light gray: full-term) for each region. See Supplementary Table S1 for region abbreviations. Source data are provided as a Source Data file.

Fig. S3: Correlation of cortical thickness centile estimates between the Bethlehem-framework and the Rutherford-framework

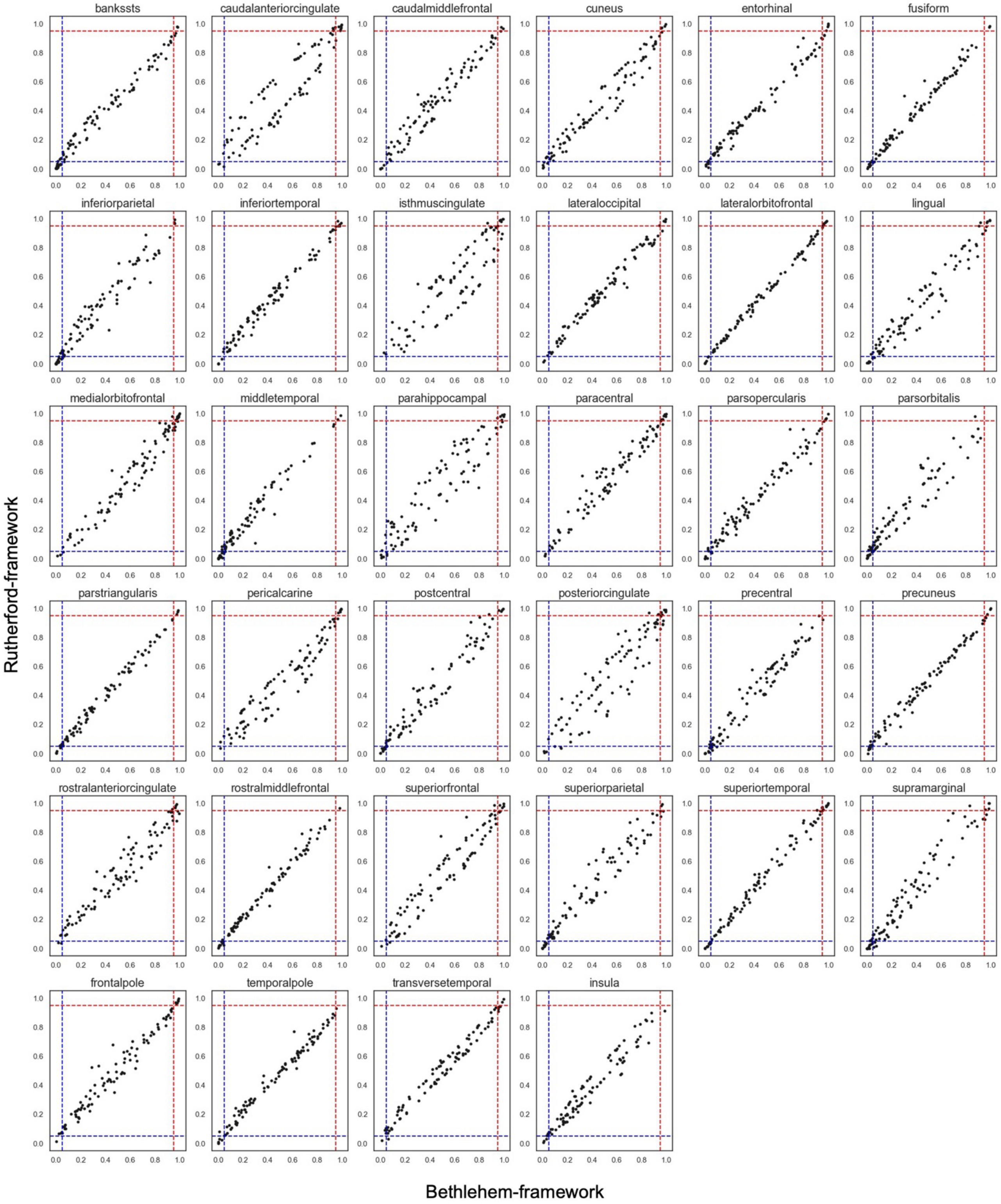

Supplementary Figure S3: Correlation of individual cortical thickness deviations estimated with two different population-based normative models. Individual deviation scores for each preterm and full-term participant in the BLS-26 cohort were estimated using an alternative normative model (Rutherford-framework, see Supplementary Methods and Results S2.2)[2]. Deviation scores estimated within the Rutherford-framework are plotted against those from the main

analysis, which used a pretrained model by Bethlehem et al. for deviation score estimation (Bethlehem-framework)[3]. Centile scores from the Bethlehem-framework are shown on the x-axis, and those from the Rutherford-framework on the y-axis. Each point represents an individual's centile estimate from both models. The blue and red dashed lines indicate the 5th and 95th percentiles, respectively, for both frameworks (horizontal lines: Rutherford-framework; vertical lines: Bethlehem-framework). Plotted Spearman correlation coefficients as well as exact p-values for each region are presented in Supplementary Table S3a. See Supplementary Table S1 for region abbreviations.

Fig. S4: Correlation of surface area centile estimates between the Bethlehem-framework and the Rutherford-framework

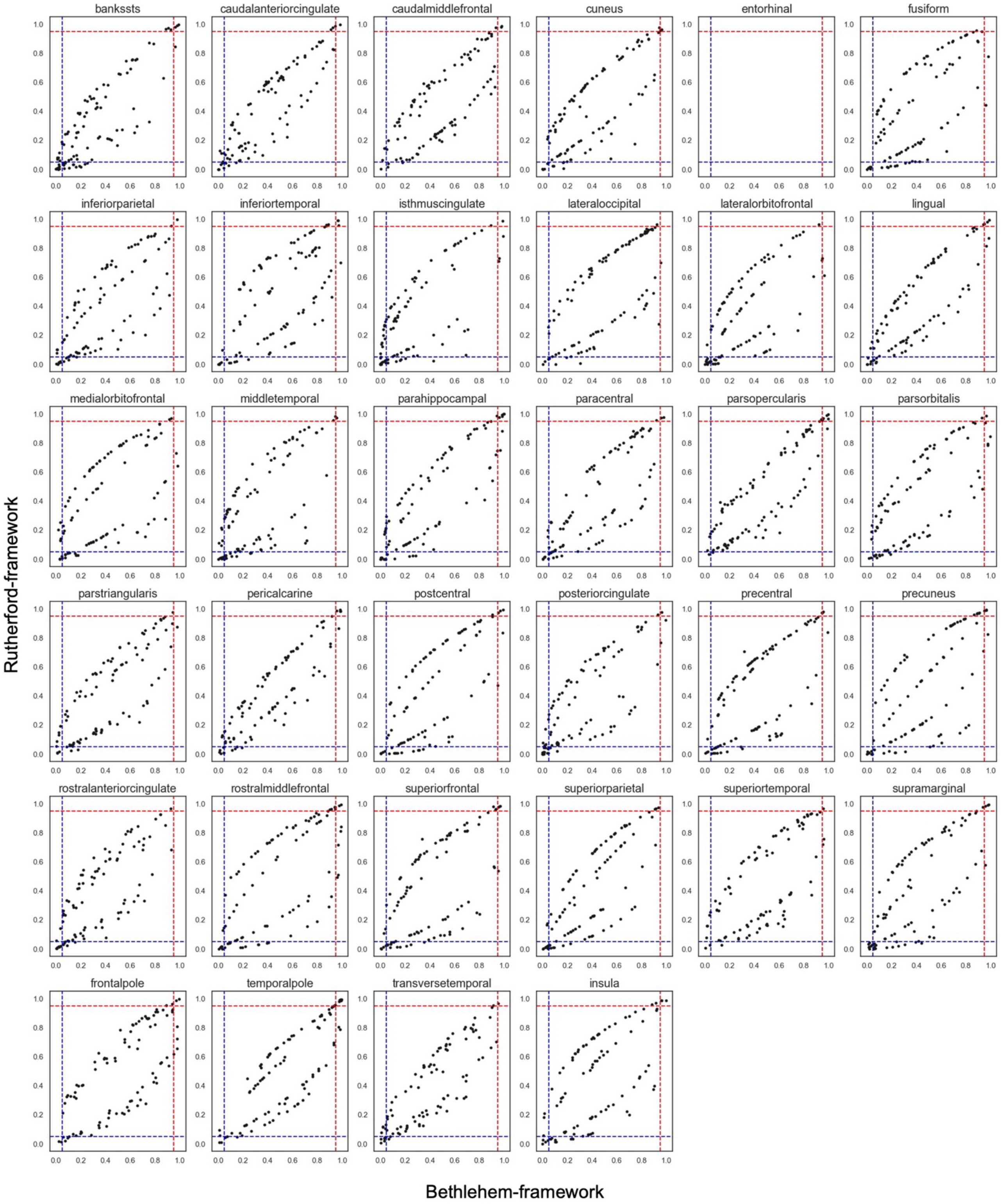

Supplementary Figure S4: Correlation of individual surface area deviations estimated with two different population-based normative models. Individual deviation scores for each preterm and full-term participant in the BLS-26 cohort were estimated using an alternative normative model (Rutherford-framework)[2]. These scores are plotted against those from the main analysis, which used a pretrained model by Bethlehem et al. for deviation score estimation (Bethlehem-framework)[3].

Centile scores from the Bethlehem-framework are shown on the x-axis, and those from the Rutherford-framework on the y-axis. Each point represents an individual's centile estimate from both models. The blue and red dashed lines indicate the 5th and 95th percentiles, respectively, for both frameworks (horizontal lines: Rutherford-framework; vertical lines: Bethlehem-framework). Plotted Spearman correlation coefficients as well as exact p-values for each region are presented in Supplementary Table S3b. See Supplementary Table S1 for region abbreviations.

Fig. S5: Spatial heterogeneity of cortical thickness individual brain abnormality patterns between preterm subjects

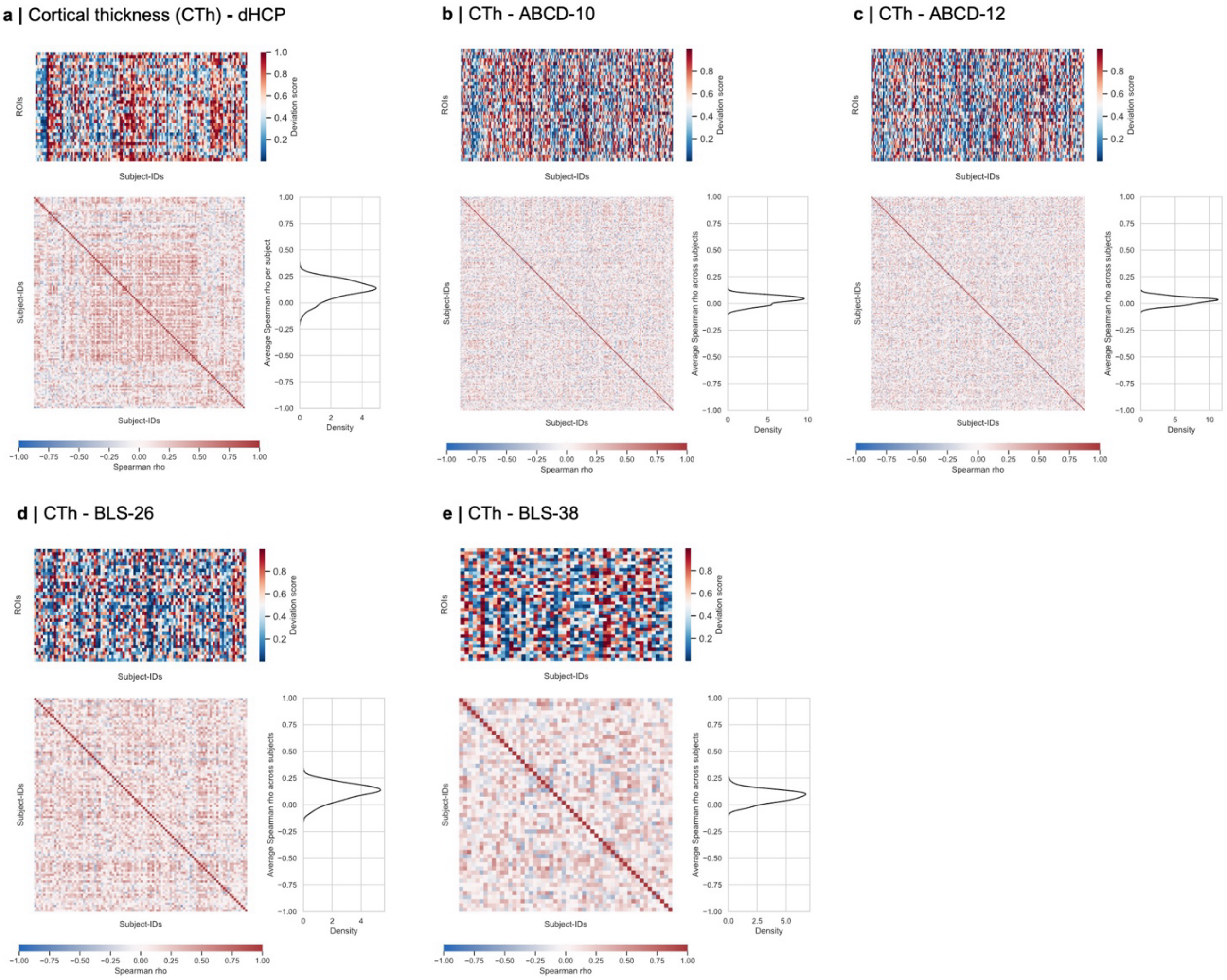

Supplementary Figure S5: Assessment of cortical thickness deviation spatial heterogeneity between preterm subjects using non-binarized deviation score profiles. Upper, Representation of cortical thickness (CTh) deviation score profiles for each preterm subject. **Lower,** Spearman correlation matrix of deviation scores across subjects with the distribution of averaged correlation coefficients for each subject with all others plotted on the side. Results are shown for dHCP (**a**), ABCD-10 (**b**), ABCD-12 (**c**), BLS-26 (**d**), and BLS-38 (**e**). Source data are provided as a Source Data file.

<u>Fig. S6: Spatial heterogeneity of surface area individual brain abnormality patterns between preterm subjects</u>

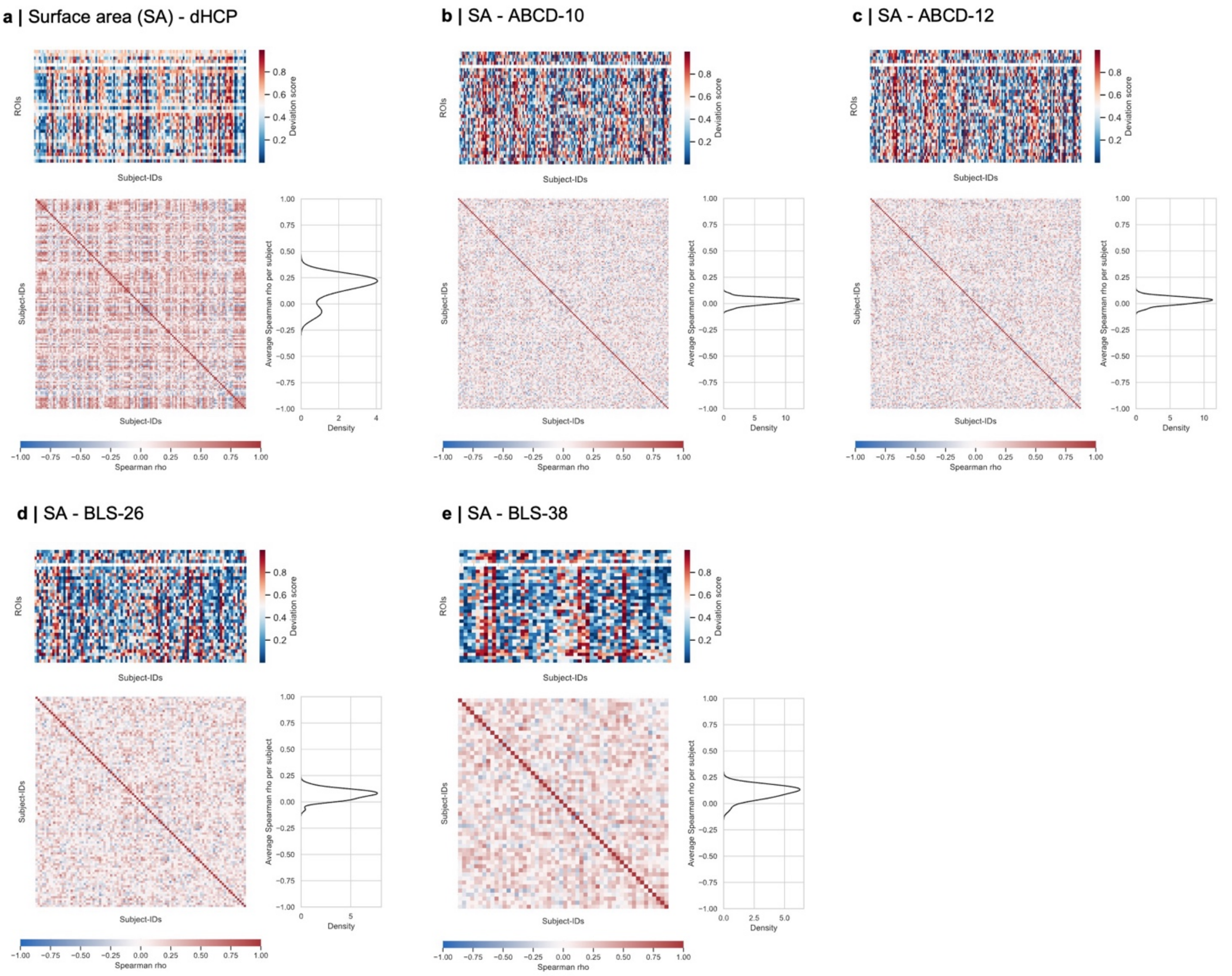

Supplementary Figure S6: Assessment of surface area deviation spatial heterogeneity between preterm subjects using non-binarized deviation score profiles. Upper, Representation of surface area (SA) deviation score profiles for each preterm subject. **Lower,** Spearman correlation matrix of deviation scores across subjects with the distribution of averaged correlation coefficients for each subject with all others plotted on the side. Results are shown for dHCP (**a**), ABCD-10 (**b**), ABCD-12 (**c**), BLS-26 (**d**), and BLS-38 (**e**). Source data and are provided as a Source Data file.

Fig. S7: Similarity of inter-regional deviation patterns of cortical thickness

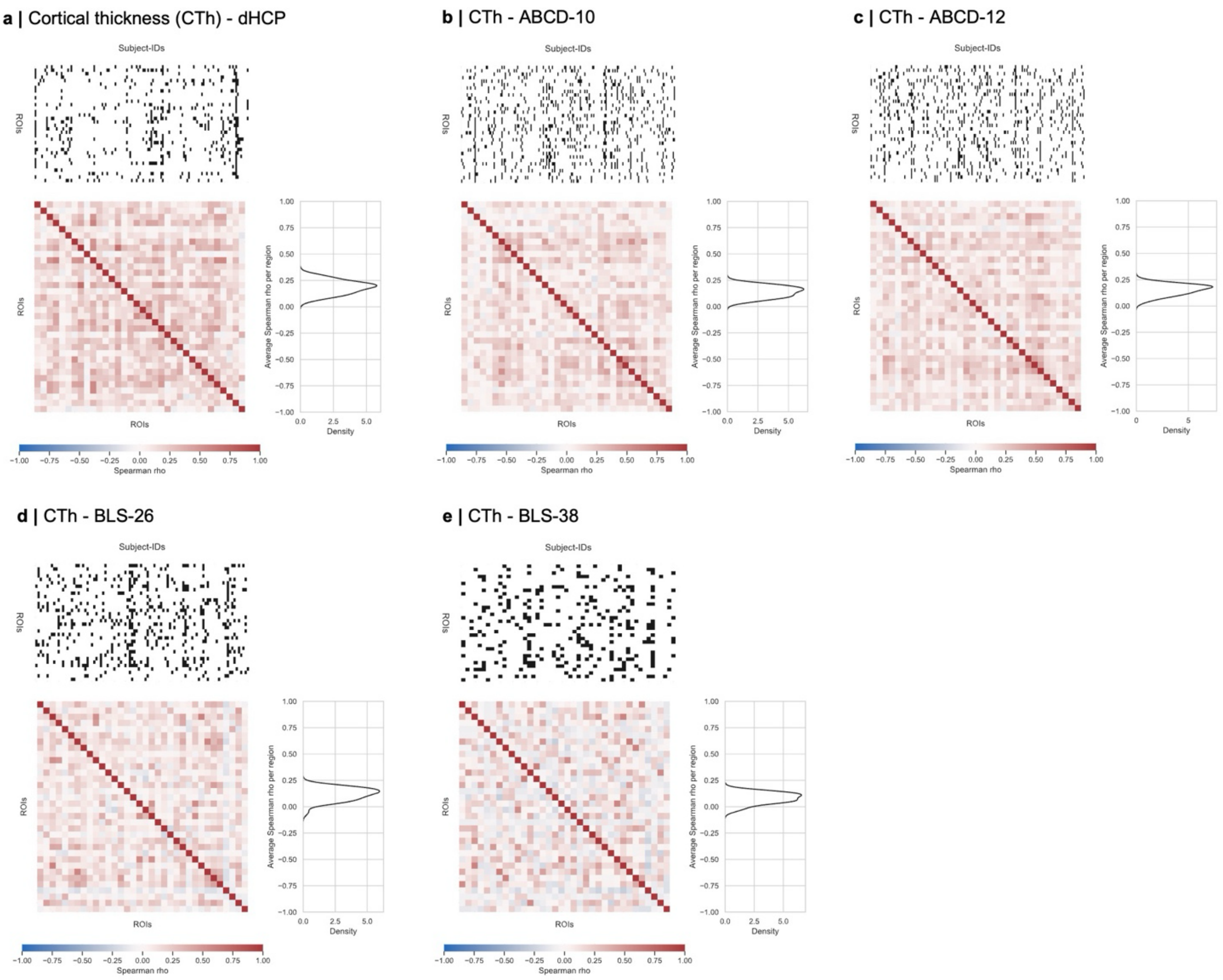

Supplementary Figure S7: Similarity of inter-regional deviation patterns of cortical thickness. Upper, Extranormal deviations were binarized (i.e., infra- and supranormal deviations are designated with 1, all others with 0). **Lower**, Spearman correlation coefficients between binarized extranormal deviations were then computed for each brain region pair across preterm subjects. The distribution of average Spearman coefficients for each region are depicted on the side. Results are shown for dHCP (**a**), ABCD-10 (**b**), ABCD-12 (**c**), BLS-26 (**d**), and BLS-38 (**e**). Source data are provided as a Source Data file.

Fig. S8: Similarity of inter-regional deviation patterns of surface area

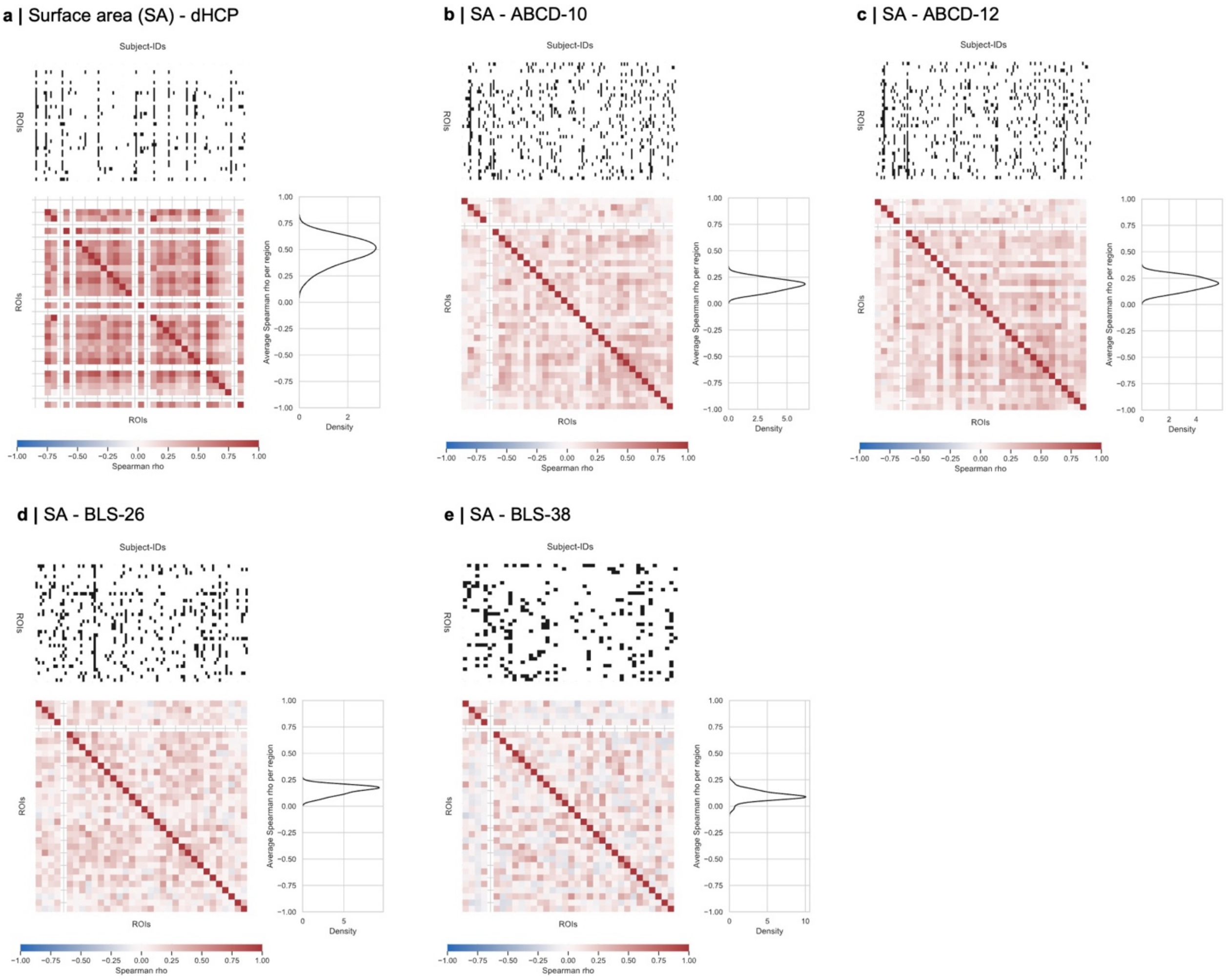

Supplementary Figure S8: Similarity of inter-regional deviation patterns of surface area. Upper, Extranormal deviations were binarized (i.e., infra- and supranormal deviations are designated with 1, all others with 0). **Lower,** Spearman correlation coefficients between binarized extranormal deviations were then computed for each brain region pair across preterm subjects. The distribution of average Spearman coefficients for each region are depicted on the side. Results are shown for dHCP (**a**), ABCD-10 (**b**), ABCD-12 (**c**), BLS-26 (**d**), and BLS-38 (**e**). Source data are provided as a Source Data file.

Fig. S9: Individual heterogeneity of cerebral tissue volume measures after preterm birth

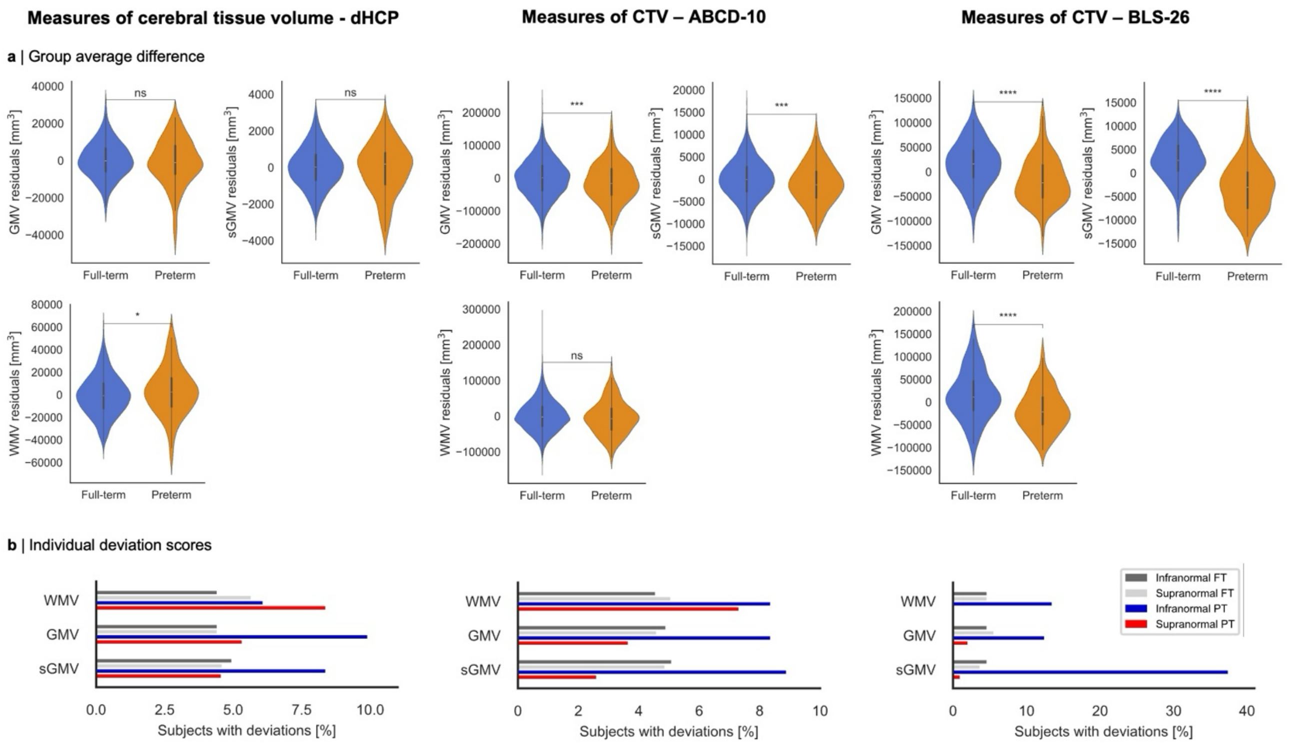

Supplementary Figure S9: Individual heterogeneity of cerebral tissue volume measures after preterm birth. The measures white matter volume (WMV), gray matter volume (GMV), and subcortical GMV (sGMV) are collectively referred to as measures of cerebral tissue volume. **a,** Group average difference of GMV, sGMV, and WMV for preterm and full-term subjects estimated by linear regression models correcting for age and sex ($p_{FDR} < 0.05$, two-sided). Residuals of cerebral tissue volume measures (i.e., with sex, age, and estimated total intracranial volume regressed out) are shown for visualization purposes. Exact T-statistics and p-values for each measure are provided in Supplementary Tables S2**. b,** Bar plots represent the percentage of preterm (PT) and full-term (FT) subjects sharing an extranormal deviation for the given cerebral tissue volume measure (infranormal: < 5th percentile, blue: preterm, dark gray: full-term; supranormal: > 95th percentile, red: preterm, light gray: full-term). Results are shown for neonates (dHCP, left), children (ABCD-10, middle), and adults (BLS-26, right). Source data are provided as a Source Data file.

Fig. S10: Relationship between cortical thickness individual brain abnormality patterns and gestational age

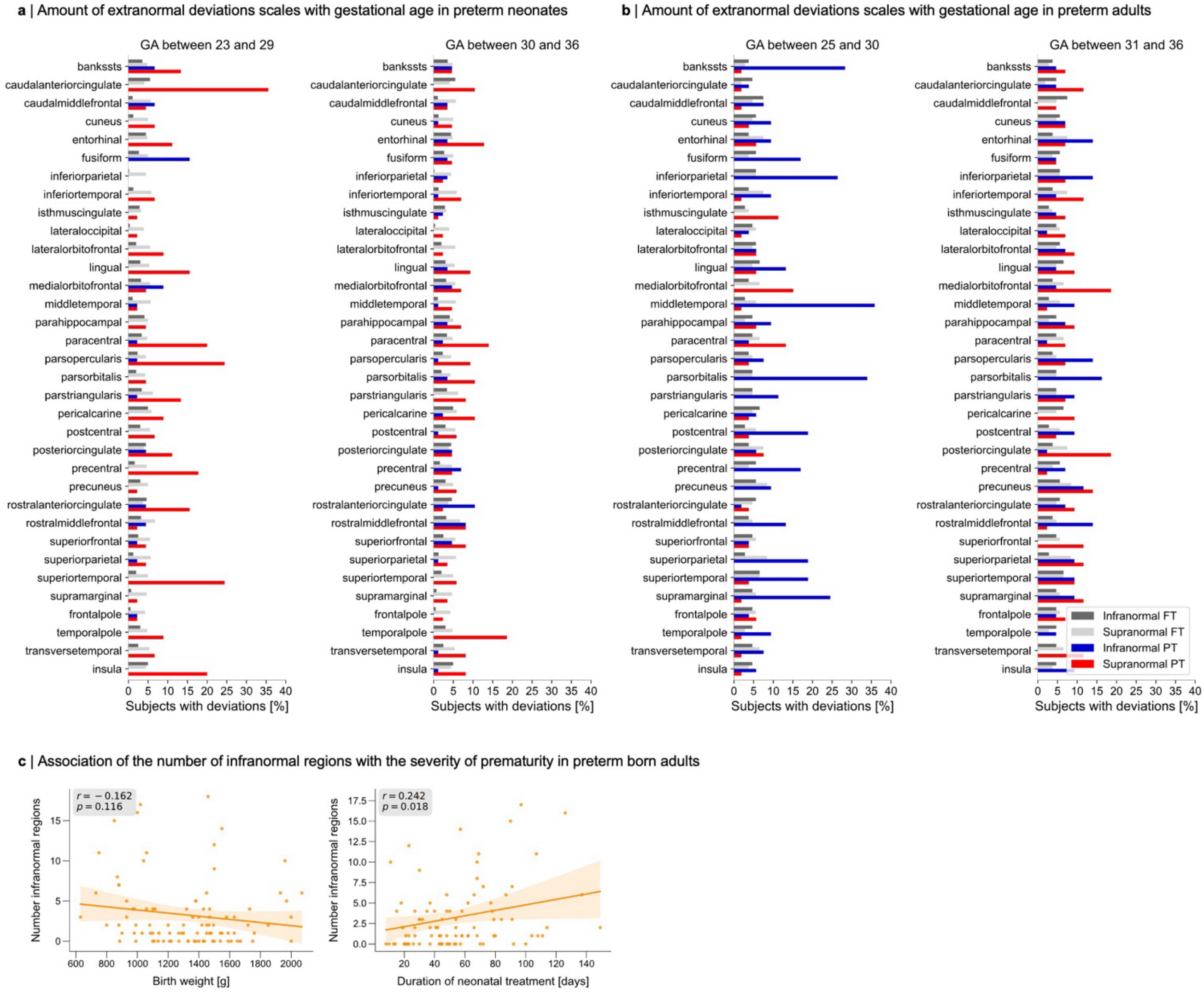

Supplementary Figure S10: Relationship between cortical thickness individual brain abnormality patterns and gestational age. a, Preterm individuals of the dHCP cohort were divided into two groups based on gestational age (GA): those born before (n = 27) and after (n = 65) the 30th week of gestation. For each group, we computed the percentage of subjects with extranormal deviations of cortical thickness in any of 34 regions (infranormal: < 5th percentile, blue: preterm, dark gray: full-term, supranormal: > 95th percentile, red: preterm, light gray: full-term). **b,** A similar analysis was conducted for preterm individuals of BLS-26, divided into earlier (GA ≤ 30 weeks, n = 53) and later preterm birth (GA > 30 weeks, n = 43) groups. Up to 36 % of earlier-born individuals showed infranormal deviations in any given region, compared to only up to 19 % of subjects born later in the preterm period. **c,** In the BLS-26 cohort, the number of infranormal cortical thickness regions per subject was correlated with measures of the severity of prematurity: birth weight (BW) and the Duration of Neonatal Treatment Index (DNTI), using Spearman's rank correlation. A significant positive correlation was found with DNTI (Spearman rho(94) = 0.242, p = 0.018; p_{FDR} = 0.036, CI = [0.043, 0.422], two-tailed), but not with BW (Spearman rho(94) = -0.162, p = 0.116, p_{FDR} = 0.116, CI = [-0.351, 0.040], two-tailed). See Supplementary Table S1 for region abbreviations. Source data are provided as a Source Data file.

Fig. S11: Relationship between cortical thickness individual brain abnormality patterns and gestational age in different stages of prematurity

a | Amount of extranormal deviations scales with gestational age in preterm neonates

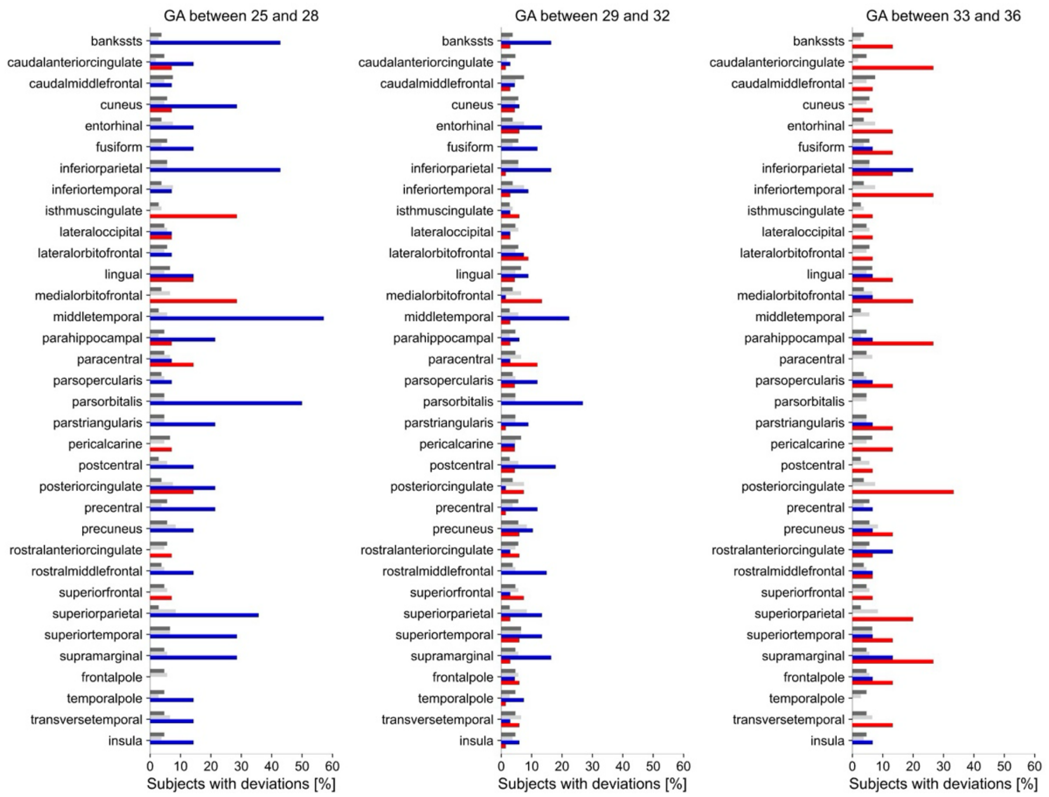

b | Amount of extranormal deviations scales with gestational age in preterm adults

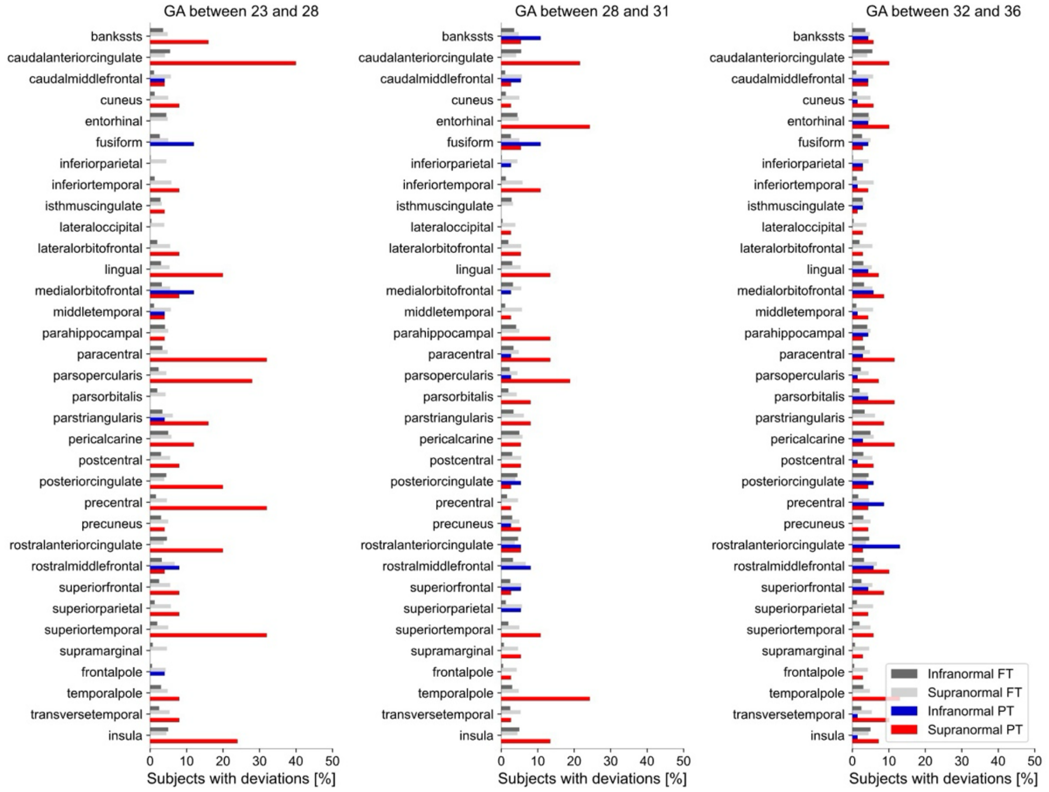

Supplementary Figure S11: Control analyses of cortical thickness deviations using finer gestational age subgrouping. a, Preterm neonates from the dHCP cohort were stratified into three groups by gestational age (GA): (i) extreme preterm (GA ≤ 28 weeks, n = 14), (ii) very preterm (28 < GA ≤ 32 weeks, n = 30), and (iii) late preterm (GA > 32 weeks, n = 48). The percentage of subjects with an extranormal deviation of regional cortical thickness (infranormal: < 5th percentile, supranormal: > 95th percentile) is shown in blue and red, respectively. Up to 57 %, 20 %, and 19 % of subjects share a supranormal deviation in the respective groups. **b,** The same analysis was applied to preterm adults of the BLS-26 cohort, with subgroup sizes of n = 14 (extreme), 67 (very), and 15 (late). Corresponding maxima for infranormal deviations in any region were 57 %, 27 %, and 20 %. See Supplementary Table S1 for region abbreviations. Source data are provided as a Source Data file.

Fig. S12: Anatomical lesion consistency of individual deviations in cortical thickness from 10 to 12 years

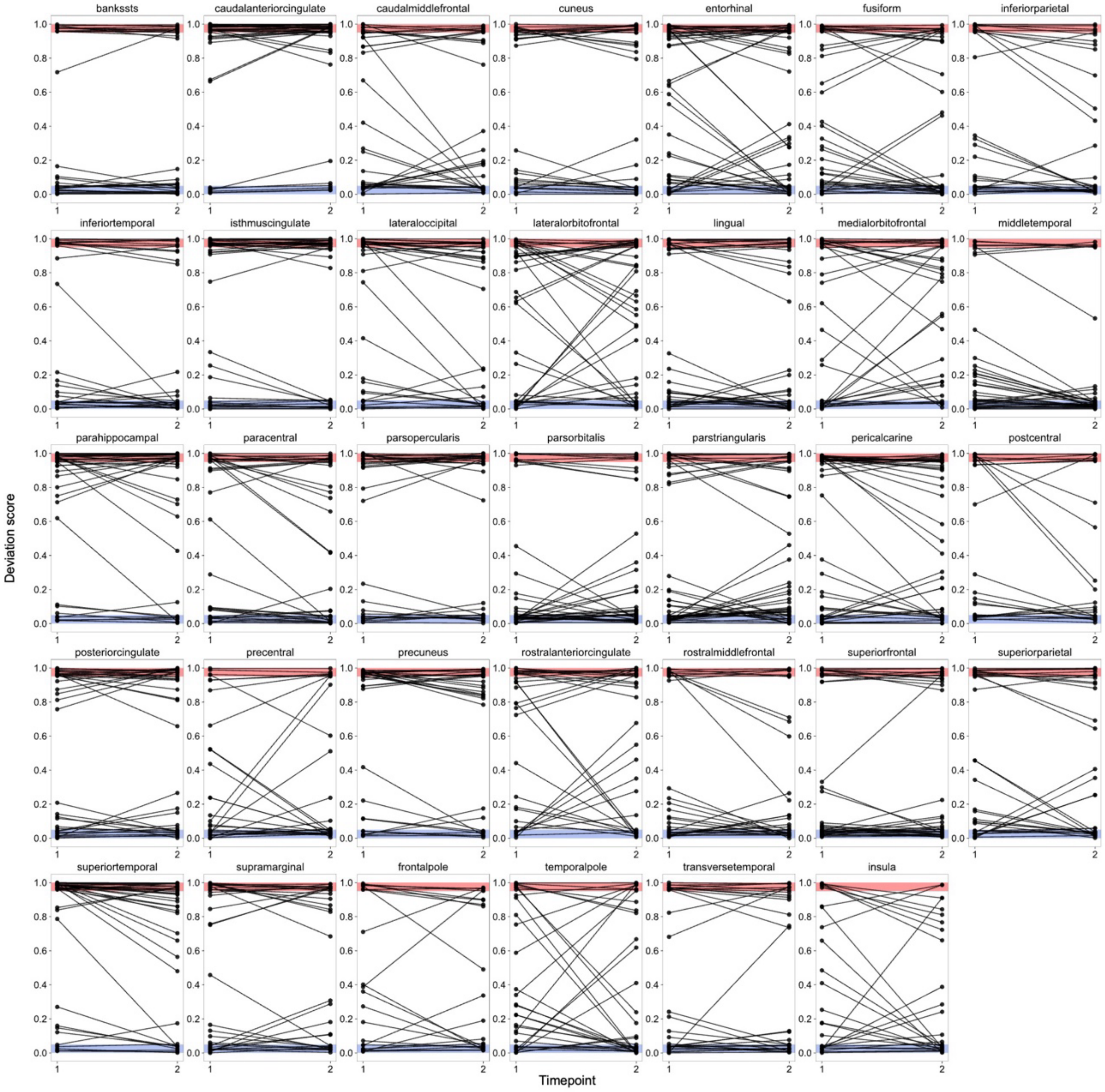

Supplementary Figure S12: Anatomical lesion consistency of individual deviations in cortical thickness from 10 to 12 years. Longitudinal deviation scores of preterm children from the ABCD Study (i.e., acquired at the ages of 10 and 12 years, n = 191) were calculated for regional cortical thickness (CTh) in the bilateral Desikan-Killiany parcellation. Only deviation scores of subjects that showed an extranormal deviation at either timepoint are depicted. See Supplementary Table S1 for region abbreviations. Source data are provided as a Source Data file.

Fig. S13: Anatomical lesion consistency of individual deviations in cortical thickness from 26 to 38 years

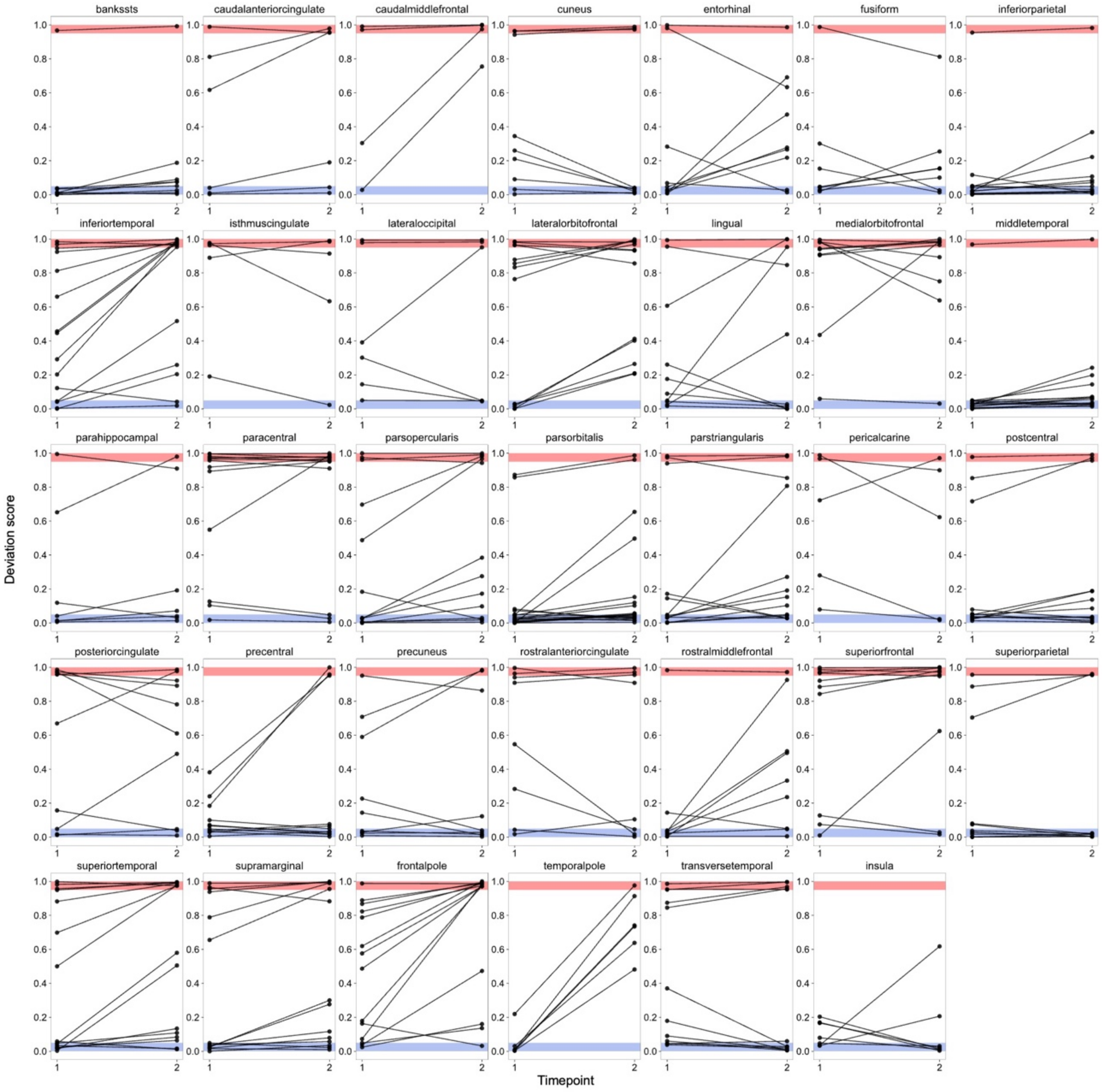

Supplementary Figure S13: Anatomical lesion consistency of individual deviations in cortical thickness from 26 to 38 years. Deviation scores of preterm adults with longitudinal data (i.e., acquired at the age of 26 and partly again at the age of 38 years, n = 46) were calculated for regional cortical thickness. Only deviation scores of subjects that showed an extranormal deviation at either timepoint are depicted. See Supplementary Table S1 for region abbreviations. Source data are provided as a Source Data file.

Fig. S14: Anatomical lesion consistency of individual deviations in surface area from 10 to 12 years

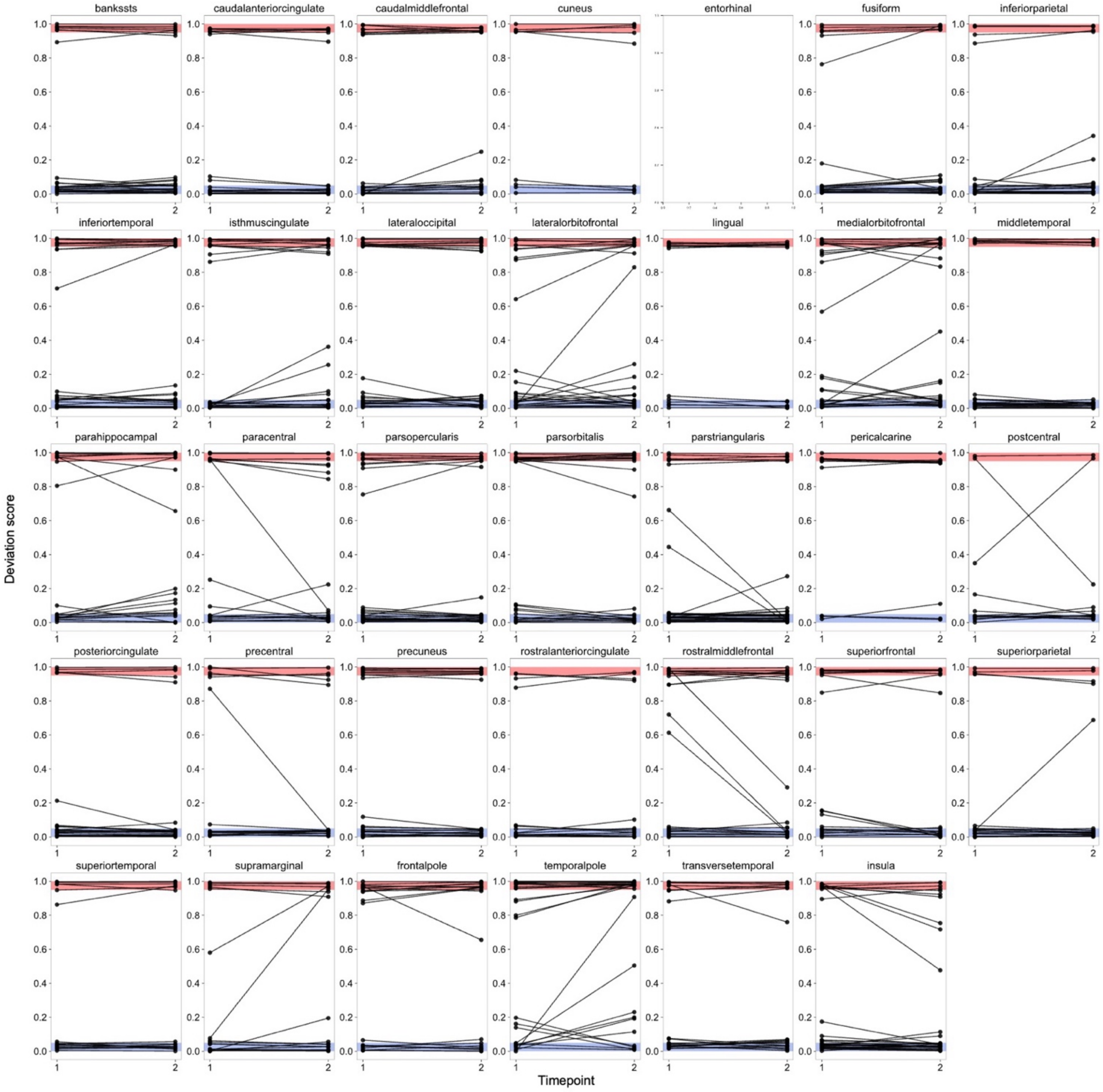

Supplementary Figure S14: Anatomical lesion consistency of individual deviations in surface area from 10 to 12 years. Extension to Fig. 6a. Longitudinal deviation scores of preterm children from the ABCD Study (n = 148) acquired at the ages of 10 and 12 years were calculated for regional surface area in the bilateral Desikan-Killiany parcellation. Only deviation scores of subjects that showed an extranormal deviation at either timepoint are depicted. Estimations for the entorhinal cortex were not possible due to missing information in the BrainChart reference models. Within-subject comparison between the two timepoints of data acquisition demonstrates that the anatomical location of infranormal (blue) and supranormal (red) deviations after preterm birth mostly remain consistent along childhood (Supplementary Table S4a). See Supplementary Table S1 for region abbreviations. Source data are provided as a Source Data file.

Fig. S15: Anatomical lesion consistency of individual deviations in surface area from 26 to 38 years

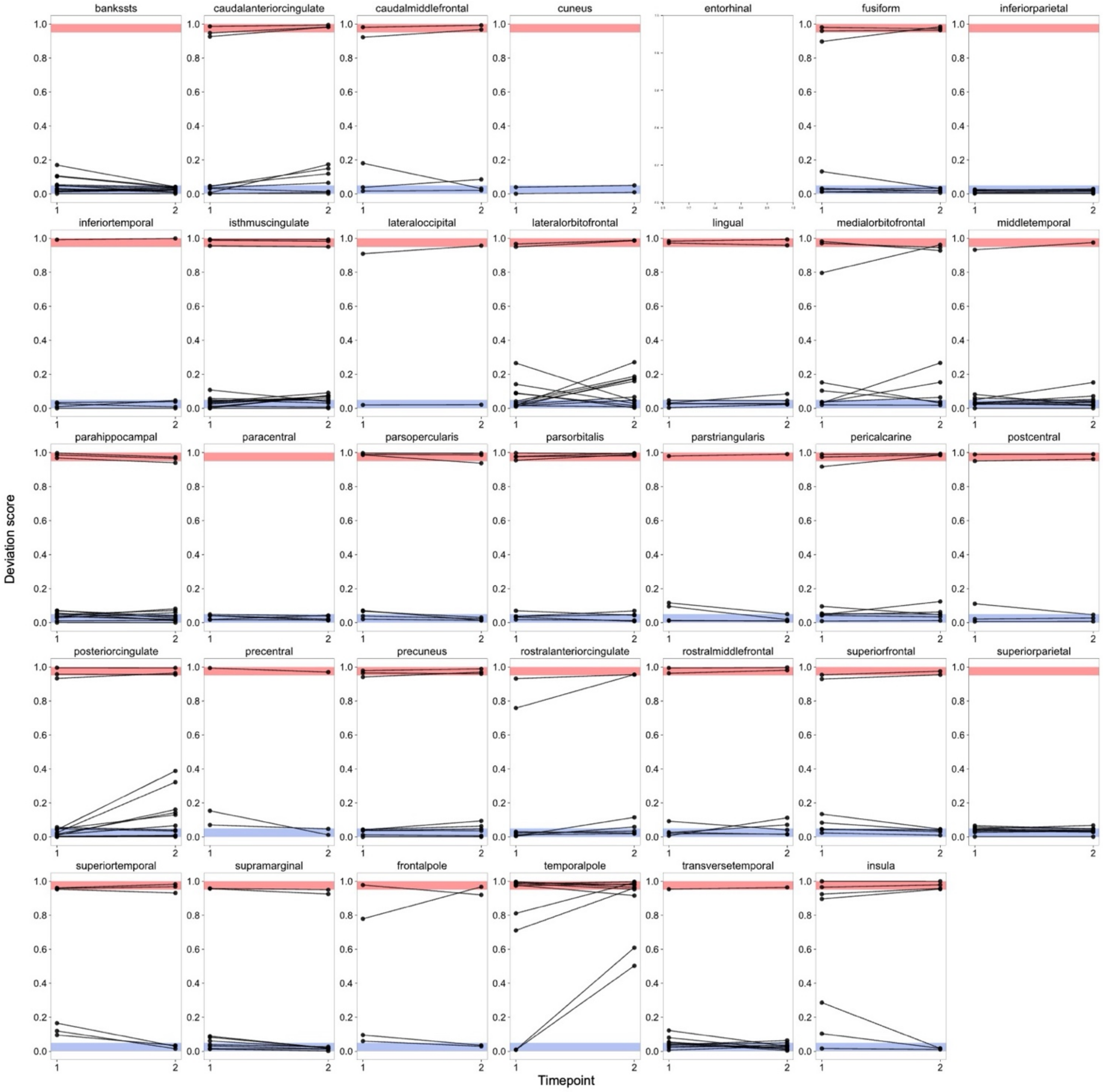

Supplementary Figure S15: Anatomical lesion consistency of individual deviations in surface area from 26 to 38 years. Extension to Fig. 4d. Deviation scores of preterm adults with longitudinal data (i.e., acquired at the age of 26 and partly again at the age of 38 years, n = 46) were calculated for regional surface area (SA). Only deviation scores of subjects that showed an extranormal deviation at either timepoint are depicted. Estimations for the entorhinal cortex were not possible due to missing information in the BrainChart reference models. Within-subject comparison between the two timepoints of data acquisition demonstrates that the anatomical location of infranormal (blue) and supranormal (red) deviations after preterm birth mostly remain consistent along adulthood (Supplementary Table S4b). See Supplementary Table S1 for region abbreviations. Source data are provided as a Source Data file.

Fig. S16 Regional distribution of Intraclass Correlation Coefficients for longitudinal cortical thickness deviations

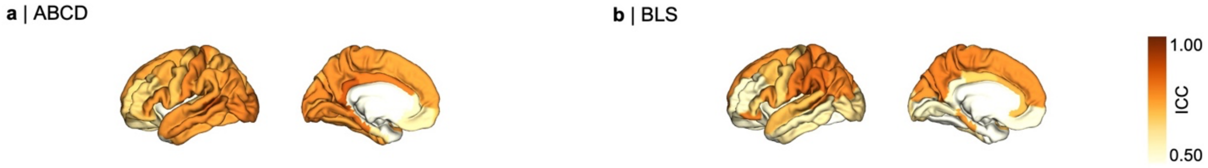

Supplementary Figure S16: Regional distribution of Intraclass Correlation Coefficients for longitudinal cortical thickness deviations. Longitudinal consistency of cortical thickness deviations was estimated using Intraclass Correlation Coefficients (ICCs). Deviation scores were calculated for both acquisitions, and ICCs were calculated across subjects for each brain region of the 34 bilateral Desikan-Killiany parcellation: for preterm children of the ABCD cohort at ages 10 and 12 years and for preterm adults of the BLS cohort at ages 26 and 38 years. Regional variations of ICC are shown on cortical surfaces of the left hemisphere for (**a**) preterm children and (**b**) preterm adults. Source Data as well as exact p-values are depicted in Supplementary Tables S4c-d.

Fig. S17: Significant associations between individual cortical thickness deviations of preterm adults and mean expression profile of eight brain cell types

a | Associations filtered for significance (p_{spin} < 0.05)

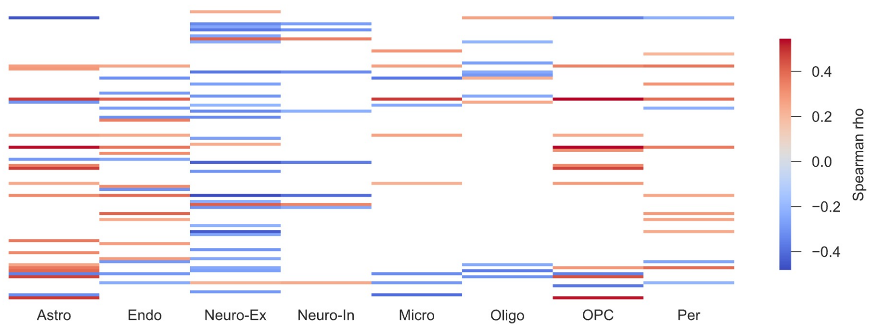

b | Associations filtered for significance (p_{SMASH} < 0.05)

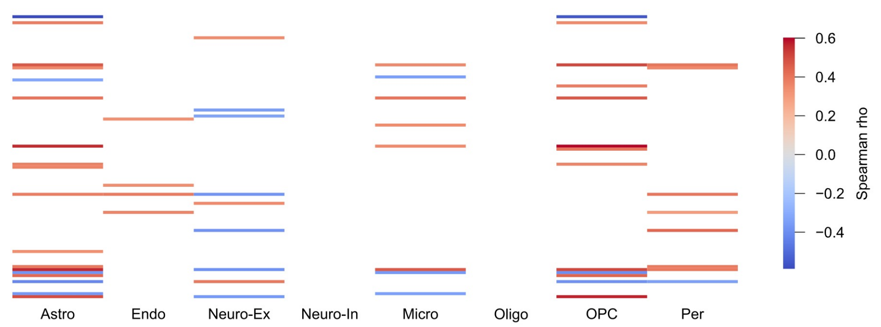

Supplementary Figure S17: Significant associations between individual cortical thickness deviations of preterm adults and mean expression profile of eight brain cell types. Spatial Spearman correlations between individual cortical thickness deviations and regional cell type abundance are presented. In comparison to Fig. 7a, this Figure only shows significant associations (p_{spin} or p_{SMASH} < 0.05). Statistical significance was assessed with two different spatial autocorrelation preserving null models, namely **(a)** spin tests (see Methods) and **(b)** brainSMASH (see Supplementary Methods). Astro astrocytes, Endo endothelial cells, Micro microglia, Neuro-Ex excitatory neurons, Neuro-In inhibitory neurons, Oligo oligodendrocytes, OPC oligodendrocyte progenitor cells, Per pericytes. Source data and exact p-values are provided as a Source Data file.

Fig. S18: Principal component loadings

a | dHCP

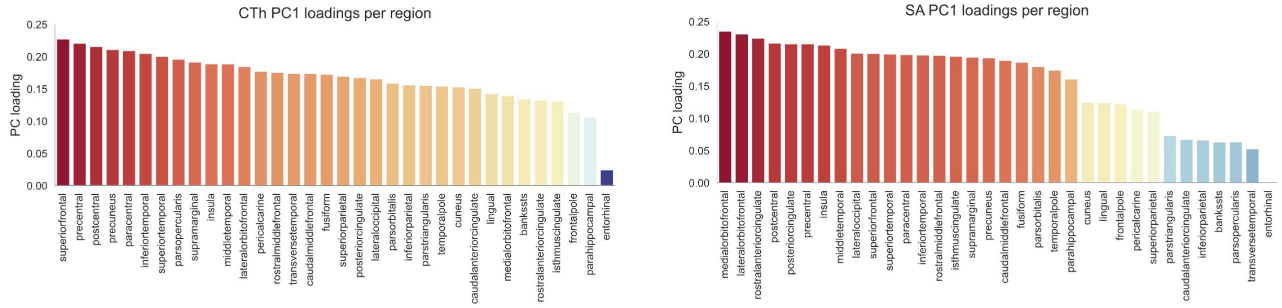

b | ABCD-10

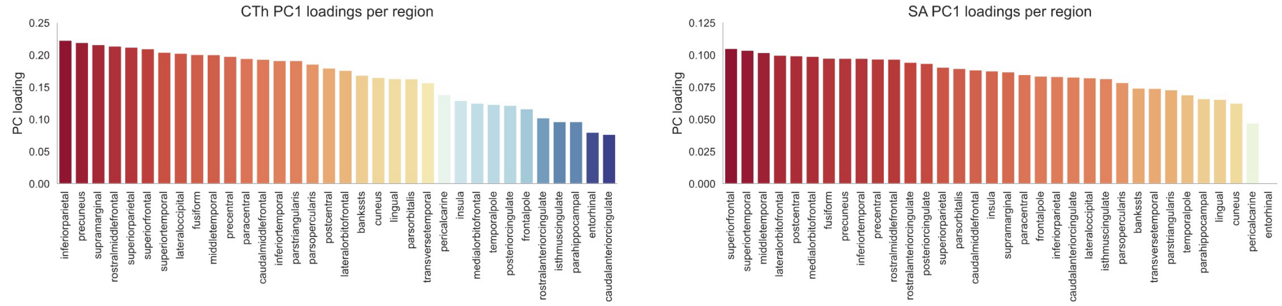

c | BLS-26

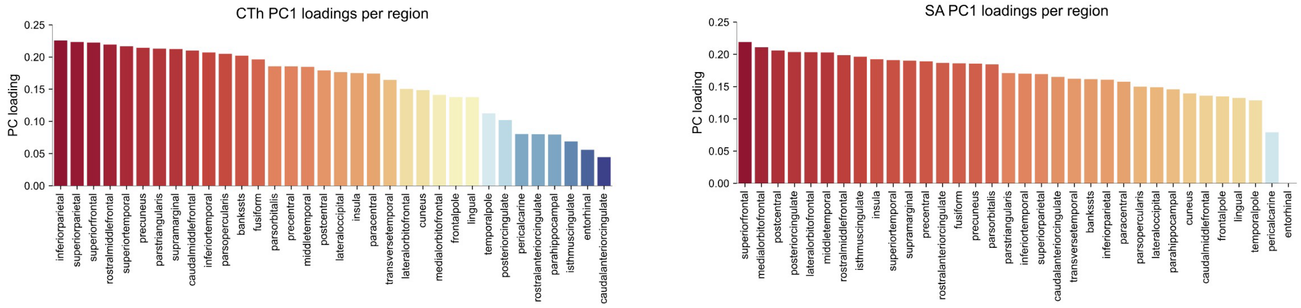

Supplementary Figure S18: Principal component loadings. Loadings of the first principal component (PC1) from Principal Component Analysis across 34 cortical regions for cortical thickness (CTh, left) and surface area (SA, right) for the (**a**) dHCP, (**b**) ABCD-10, and (**c**) BLS-26 cohorts. Cortical region's PC loadings are ordered from highest to lowest. See Supplementary Table S1 for region abbreviations. Source data are provided as a Source Data file.

Fig. S19: Control analyses for the associations between the first principal component across regional cortical thickness and socio-economic status

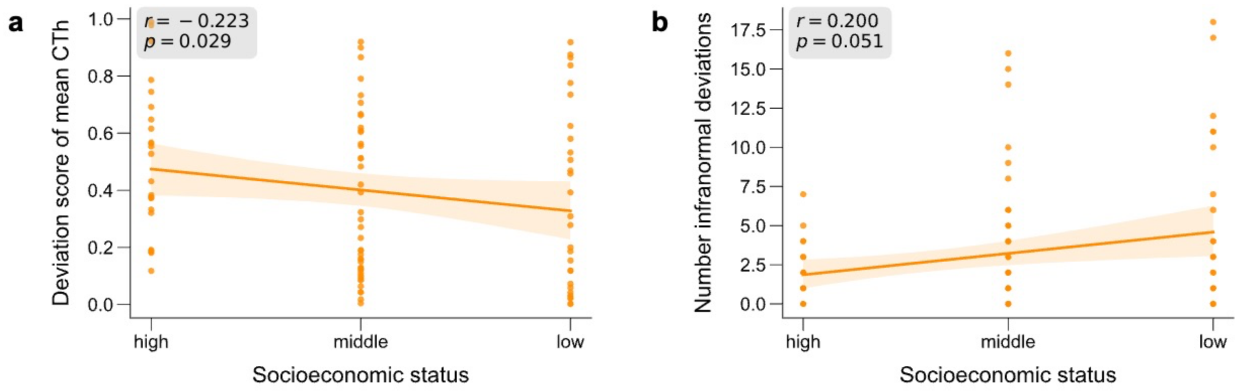

Supplementary Figure S19: Control analyses for the associations between the first principal component across regional cortical thickness and socio-economic status. Instead of the principal component 1 (PC1) across regional cortical thickness (CTh, see Fig. 8b), the deviation scores of mean CTh across all 34 cortical regions (**a,** Spearman rho(94) = -0.223, p = 0.029, p_{FDR} = 0.051, CI = [-0.405,-0.023], two-tailed) as well as the number of regions with infranormal CTh per subject (**b,** Spearman rho(94) = 0.200, p = 0.051, p_{FDR} = 0.051, CI = [0.000,0.385], two-tailed) were used for Spearman correlation analysis with socio-economic status (SES). Data of preterm adults from the BLS-26 cohort were used. Source data are provided as a Source Data file.

Fig. S20: Control analyses for the associations between the first principal component across regional cortical thickness and IQ

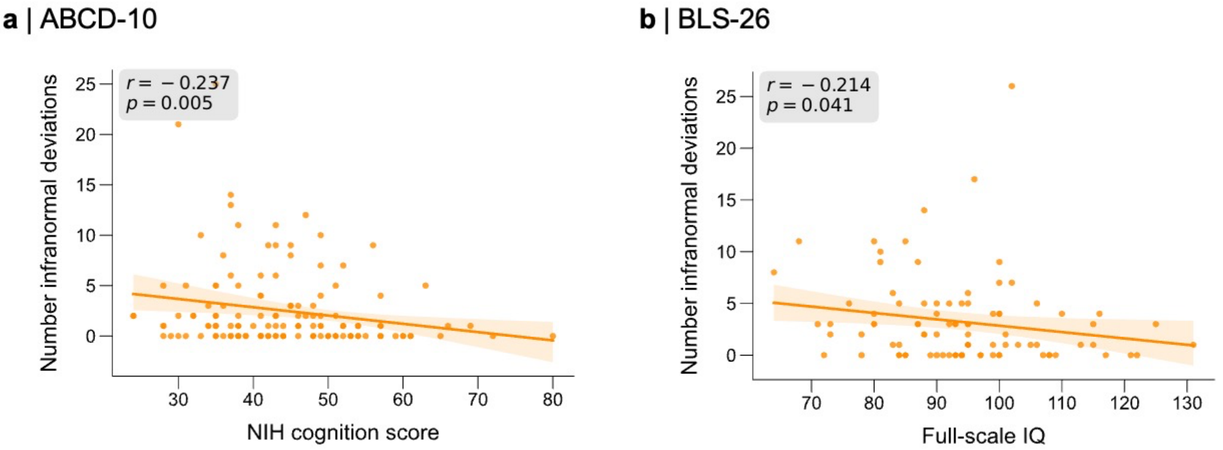

Supplementary Figure S20: Control analyses for the associations between the first principal component across regional cortical thickness and IQ. Instead of the principal component 1 (PC1) across surface area (SA, see Fig. 9), the number of regions with infranormal SA per subject were used for Spearman correlation analysis with measures of cognitive performance. Results are shown for preterm children of the ABCD-10 cohort (**a**) and preterm adults of the BLS-26 cohort (**b**). Source data are provided as a Source Data file.

4. Supplementary Tables

Table S1: Regions of the Desikan-Killiany atlas[25]

Region	Abbreviation used	Number in Fig. 3 and 4
Banks superior temporal sulcus	bankssts	1
Caudal anterior-cingulate cortex	caudalanteriorcingulate	2
Caudal middle frontal gyrus	caudalmiddlefrontal	3
Cuneus cortex	cuneus	4
Entorhinal cortex	entorhinal	5
Fusiform gyrus	fusiform	6
Inferior parietal cortex	inferiorparietal	7
Inferior temporal gyrus	inferiortemporal	8
Isthmus– cingulate cortex	isthmuscingulate	9
Lateral occipital cortex	lateraloccipital	10
Lateral orbital frontal cortex	lateralorbitofrontal	11
Lingual gyrus	lingual	12
Medial orbital frontal cortex	medialorbitofrontal	13
Middle temporal gyrus	middletemporal	14
Parahippocampal gyrus	parahippocampal	15
Paracentral lobule	paracentral	16
Pars opercularis	parsopercularis	17
Pars orbitalis	parsorbitalis	18
Pars triangularis	parstriangularis	19
Pericalcarine cortex	pericalcarine	20
Postcentral gyrus	postcentral	21
Posterior-cingulate cortex	posteriorcingulate	22
Precentral gyrus	precentral	23
Precuneus cortex	precuneus	24
Rostral anterior cingulate cortex	rostralanteriorcingulate	25
Rostral middle frontal gyrus	rostralmiddlefrontal	26
Superior frontal gyrus	superiorfrontal	27
Superior parietal cortex	superiorparietal	28
Superior temporal gyrus	superiortemporal	29
Supramarginal gyrus	supramarginal	30
Frontal pole	frontalpole	31
Temporal pole	temporalpole	32
Transverse temporal cortex	transversetemporal	33
Insular cortex	insula	34

Table S2: Statistics for average dysmaturation outcomes

See Supplementary Table S1 for region abbreviations. Two-sided p-values (uncorrected as well as corrected for the False Discovery Rate, FDR) are provided in the following.

Table S2a: Average dysmaturation outcomes of cortical thickness in the dHCP cohort

ROI	T_{691}	Cohen's d	95% CI lower	95% CI upper	p-value	p_{FDR}
bankssts	1.255	-0.111	-0.005	0.022	0.210	0.252
caudalanteriorcingulate	9.736	-0.741	0.048	0.072	**4.44E-21**	**7.55E-20**
caudalmiddlefrontal	-0.991	0.068	-0.018	0.006	0.322	0.365
cuneus	3.696	-0.313	0.010	0.031	**2.37E-04**	**5.03E-04**
entorhinal	3.604	-0.363	0.019	0.065	**3.36E-04**	**6.73E-04**
fusiform	-0.627	0.051	-0.013	0.007	0.531	0.564
inferiorparietal	-2.707	0.273	-0.027	-0.004	**6.96E-03**	**1.06E-02**
inferiortemporal	2.882	-0.274	0.005	0.027	**4.08E-03**	**7.70E-03**
isthmuscingulate	2.757	-0.044	0.003	0.020	**5.99E-03**	**1.01E-02**
lateraloccipital	1.242	-0.099	-0.004	0.019	0.215	0.252
lateralorbitofrontal	4.084	-0.344	0.011	0.032	**4.94E-05**	**1.29E-04**
lingual	4.093	-0.356	0.010	0.029	**4.76E-05**	**1.29E-04**
medialorbitofrontal	0.506	-0.010	-0.007	0.011	0.613	0.632
middletemporal	2.305	-0.208	0.002	0.023	**0.021**	**0.030**
parahippocampal	5.011	-0.279	0.022	0.051	**6.90E-07**	**2.13E-06**
paracentral	7.597	-0.610	0.038	0.064	**9.88E-14**	**1.12E-12**
parsopercularis	7.174	-0.686	0.029	0.050	**1.88E-12**	**1.59E-11**
parsorbitalis	2.695	-0.267	0.004	0.028	**0.007**	**0.011**
parstriangularis	2.744	-0.272	0.004	0.027	**0.006**	**0.010**
pericalcarine	6.473	-0.446	0.028	0.053	**1.83E-10**	**1.03E-09**
postcentral	5.499	-0.393	0.016	0.035	**5.39E-08**	**1.83E-07**
posteriorcingulate	3.881	-0.168	0.009	0.028	**1.14E-04**	**2.60E-04**
precentral	5.782	-0.459	0.019	0.038	**1.12E-08**	**4.23E-08**
precuneus	3.879	-0.267	0.010	0.032	**1.15E-04**	**2.60E-04**
rostralanteriorcingulate	1.830	-0.021	-0.001	0.020	0.068	0.089
rostralmiddlefrontal	-2.843	0.157	-0.027	-0.005	**0.005**	**0.008**
superiorfrontal	2.186	-0.228	0.001	0.022	**0.029**	**0.040**
superiorparietal	-0.478	0.066	-0.014	0.008	0.633	0.633
superiortemporal	10.222	-0.810	0.044	0.065	**6.14E-23**	**2.09E-21**
supramarginal	1.441	-0.108	-0.003	0.019	0.150	0.189
frontalpole	0.941	-0.091	-0.008	0.023	0.347	0.380
temporalpole	5.988	-0.526	0.038	0.075	**3.42E-09**	**1.66E-08**
transversetemporal	5.896	-0.332	0.034	0.069	**5.83E-09**	**2.48E-08**

insula	6.942	-0.551	0.024	0.044	**8.90E-12**	**6.05E-11**

Table S2b: Average dysmaturation outcomes of surface area in the dHCP cohort

ROI	T_{691}	Cohen's d	95% CI lower	95% CI upper	p-value	p_{FDR}
bankssts	-3.224	0.426	-23.171	-5.629	**0.001**	**0.005**
caudalanteriorcingulate	0.357	0.187	-11.106	16.049	0.721	0.817
caudalmiddlefrontal	-2.069	0.343	-43.166	-1.134	**0.039**	0.066
cuneus	-0.124	0.186	-29.293	25.802	0.901	0.928
entorhinal	0.508	0.095	-3.170	5.383	0.611	0.743
fusiform	-2.843	0.395	-68.961	-12.616	**0.005**	**0.013**
inferiorparietal	-3.656	0.447	-156.793	-47.221	**2.76E-04**	**1.17E-03**
inferiortemporal	-1.207	0.291	-49.584	11.837	0.228	0.298
isthmuscingulate	-3.864	0.464	-50.936	-16.612	**1.22E-04**	**5.93E-04**
lateraloccipital	-1.683	0.316	-100.286	7.713	0.093	0.137
lateralorbitofrontal	-4.354	0.487	-136.806	-51.774	**1.54E-05**	**1.04E-04**
lingual	-1.389	0.290	-57.130	9.789	0.165	0.227
medialorbitofrontal	-4.199	0.483	-57.378	-20.813	**3.04E-05**	**1.72E-04**
middletemporal	-3.528	0.434	-101.128	-28.818	**4.46E-04**	**1.68E-03**
parahippocampal	-0.415	0.221	-8.420	5.482	0.678	0.795
paracentral	0.021	0.194	-13.056	13.343	0.983	0.983
parsopercularis	-5.131	0.544	-61.739	-27.568	**3.74E-07**	**6.36E-06**
parsorbitalis	-6.030	0.609	-39.818	-20.258	**2.67E-09**	**9.07E-08**
parstriangularis	-4.893	0.529	-38.900	-16.622	**1.23E-06**	**1.40E-05**
pericalcarine	2.331	-0.054	6.532	76.284	**0.020**	**0.040**
postcentral	-0.791	0.262	-67.588	28.757	0.429	0.540
posteriorcingulate	-0.290	0.227	-16.597	12.323	0.772	0.846
precentral	-1.383	0.298	-66.021	11.452	0.167	0.227
precuneus	-1.853	0.328	-63.979	1.846	0.064	0.099
rostralanteriorcingulate	-4.657	0.514	-42.552	-17.313	**3.85E-06**	**3.27E-05**
rostralmiddlefrontal	-2.860	0.391	-132.834	-24.682	**0.004**	**0.013**
superiorfrontal	-2.692	0.381	-193.206	-30.231	**0.007**	**0.019**
superiorparietal	-2.643	0.386	-130.999	-19.323	**0.008**	**0.020**
superiortemporal	-2.096	0.348	-76.983	-2.523	**0.036**	0.065
supramarginal	-2.377	0.365	-89.217	-8.504	**0.018**	**0.040**
frontalpole	-1.915	0.276	-9.218	0.115	0.056	0.091
temporalpole	0.188	0.173	-6.778	8.210	0.851	0.904
transversetemporal	-2.308	0.345	-13.163	-1.063	**0.021**	**0.040**
insula	-2.319	0.354	-41.660	-3.457	**0.021**	**0.040**

Table S2c: Average dysmaturation outcomes of cerebral tissue volume measures in the dHCP cohort

**ROI**	**T_{691}**	**Cohen's d**	**95% CI lower**	**95% CI upper**	**p-value**	**p_{FDR}**
WMV	1.995	-0.006	56.92	7099.351	**0.046**	0.093
GMV	-1.332	0.306	-3274.214	626.972	0.183	0.244
sGMV	-0.896	0.253	-323.871	120.898	0.371	0.371
mean CTh	5.358	-0.419	0.013	0.028	**1.15E-07**	**4.58E-07**

Table S2d: Average dysmaturation outcomes of cortical thickness in the ABCD-10 cohort

ROI	T_{5949}	Cohen's d	95% CI lower	95% CI upper	p-value	p_{FDR}
bankssts	-1.724	0.136	-0.035	0.002	0.085	0.200
caudalanteriorcingulate	2.468	-0.171	0.006	0.051	**0.014**	0.091
caudalmiddlefrontal	-1.747	0.132	-0.031	0.002	0.081	0.200
cuneus	1.621	-0.100	-0.003	0.033	0.105	0.223
entorhinal	1.742	-0.125	-0.004	0.069	0.082	0.200
fusiform	-0.664	0.062	-0.019	0.009	0.507	0.718
inferiorparietal	-1.325	0.114	-0.024	0.005	0.185	0.350
inferiortemporal	-0.211	0.024	-0.018	0.015	0.833	0.939
isthmuscingulate	2.138	-0.148	0.002	0.037	**0.033**	0.111
lateraloccipital	2.845	-0.199	0.008	0.042	**0.004**	0.051
lateralorbitofrontal	0.810	-0.044	-0.009	0.022	0.418	0.646
lingual	-0.196	0.032	-0.018	0.015	0.845	0.939
medialorbitofrontal	1.128	-0.065	-0.007	0.026	0.259	0.464
middletemporal	-4.238	0.321	-0.059	-0.022	**2.29E-05**	**7.79E-04**
parahippocampal	2.336	-0.162	0.005	0.062	**0.020**	0.091
paracentral	-0.221	0.026	-0.019	0.015	0.825	0.939
parsopercularis	1.347	-0.094	-0.005	0.026	0.178	0.350
parsorbitalis	-3.317	0.253	-0.055	-0.014	**0.001**	**0.016**
parstriangularis	-2.299	0.180	-0.034	-0.003	**0.022**	0.091
pericalcarine	2.503	-0.175	0.006	0.046	**0.012**	0.091
postcentral	0.353	-0.012	-0.015	0.022	0.724	0.939
posteriorcingulate	0.131	<0.001	-0.014	0.016	0.896	0.939
precentral	-1.079	0.076	-0.026	0.008	0.281	0.477
precuneus	1.037	-0.055	-0.006	0.021	0.300	0.485
rostralanteriorcingulate	0.111	0.004	-0.021	0.024	0.912	0.939
rostralmiddlefrontal	-2.256	0.181	-0.031	-0.002	**0.024**	0.091
superiorfrontal	-1.706	0.134	-0.030	0.002	0.088	0.200
superiorparietal	0.244	-0.002	-0.013	0.017	0.807	0.939
superiortemporal	2.292	-0.161	0.003	0.040	**0.022**	0.091
supramarginal	0.700	-0.041	-0.011	0.022	0.484	0.716
frontalpole	-0.177	0.026	-0.032	0.027	0.860	0.939
temporalpole	-0.594	0.047	-0.044	0.024	0.553	0.751
transversetemporal	0.025	0.008	-0.023	0.023	0.980	0.980
insula	-2.049	0.160	-0.039	-0.001	**0.041**	0.125

Table S2e: Average dysmaturation outcomes of surface area in the ABCD-10 cohort

ROI	T_{5073}	Cohen's d	95% CI lower	95% CI upper	p-value	p_{FDR}
bankssts	-2.544	0.208	-56.492	-7.320	**0.011**	**0.036**
caudalanteriorcingulate	-0.364	0.030	-24.150	16.587	0.716	0.761
caudalmiddlefrontal	-1.749	0.141	-116.979	6.664	0.080	0.139
cuneus	-0.814	0.070	-47.922	19.812	0.416	0.524
entorhinal	1.888	-0.147	-0.494	26.249	0.059	0.122
fusiform	-2.241	0.174	-119.737	-7.995	**0.025**	0.071
inferiorparietal	-3.462	0.278	-317.476	-87.899	**0.001**	**0.009**
inferiortemporal	0.149	-0.008	-64.273	74.859	0.881	0.881
isthmuscingulate	-3.046	0.245	-58.699	-12.728	**0.002**	**0.016**
lateraloccipital	-1.931	0.156	-199.841	1.503	0.054	0.121
lateralorbitofrontal	-2.524	0.201	-114.180	-14.341	**0.012**	**0.036**
lingual	-0.823	0.072	-94.834	38.744	0.410	0.524
medialorbitofrontal	-1.850	0.145	-67.301	1.956	0.064	0.122
middletemporal	-2.795	0.221	-170.983	-30.015	**0.005**	**0.022**
parahippocampal	-2.209	0.176	-26.591	-1.586	**0.027**	0.071
paracentral	0.252	-0.015	-24.571	31.828	0.801	0.825
parsopercularis	-2.810	0.232	-94.492	-16.833	**0.005**	**0.022**
parsorbitalis	-0.451	0.039	-20.184	12.628	0.652	0.715
parstriangularis	-3.173	0.254	-96.070	-22.698	**0.002**	**0.013**
pericalcarine	0.633	-0.050	-27.295	53.326	0.527	0.618
postcentral	-2.792	0.227	-194.354	-33.997	**0.005**	**0.022**
posteriorcingulate	-1.692	0.138	-50.278	3.691	0.091	0.147
precentral	-1.154	0.094	-133.405	34.543	0.249	0.338
precuneus	-1.524	0.125	-148.825	18.619	0.127	0.197
rostralanteriorcingulate	-1.859	0.151	-39.580	1.050	0.063	0.122
rostralmiddlefrontal	0.726	-0.053	-78.415	170.599	0.468	0.568
superiorfrontal	-1.261	0.100	-244.359	53.030	0.207	0.294
superiorparietal	-3.518	0.292	-338.014	-96.105	**4.38E-04**	**9.20E-03**
superiortemporal	-1.966	0.156	-144.527	-0.201	**0.049**	0.120
supramarginal	-1.299	0.108	-168.228	34.126	0.194	0.287
frontalpole	0.511	-0.032	-4.590	7.827	0.609	0.691
temporalpole	2.735	-0.209	3.807	23.083	**0.006**	**0.024**
transversetemporal	-1.742	0.143	-16.598	0.981	0.082	0.139
insula	-3.223	0.252	-103.510	-25.209	**0.001**	**0.013**

Table S2f: Average dysmaturation outcomes of cerebral tissue volume measures in the ABCD-10 cohort

ROI	T_{5949}	Cohen's d	95% CI lower	95% CI upper	p-value	p_{FDR}
WMV	-1.749	0.099	-11613.817	661.842	0.080	0.107
GMV	-3.429	0.225	-22918.643	-6246.422	**0.001**	**0.002**
sGMV	-3.293	0.207	-1671.250	-423.918	**0.001**	**0.002**
mean CTh	-0.372	0.043	-0.013	0.009	0.710	0.710

Table S2g: Average dysmaturation outcomes of cortical thickness in the ABCD-12 cohort

ROI	T_{5949}	Cohen's d	95% CI lower	95% CI upper	p-value	p_{FDR}
bankssts	-2.319	0.181	-0.040	-0.003	**0.020**	0.074
caudalanteriorcingulate	2.707	-0.189	0.008	0.052	**0.007**	**0.033**
caudalmiddlefrontal	-2.712	0.208	-0.039	-0.006	**0.007**	**0.033**
cuneus	0.753	-0.042	-0.011	0.024	0.452	0.640
entorhinal	-0.011	0.003	-0.036	0.035	0.992	0.992
fusiform	-1.480	0.119	-0.025	0.003	0.139	0.295
inferiorparietal	-2.237	0.179	-0.031	-0.002	**0.025**	0.078
inferiortemporal	-1.500	0.118	-0.029	0.004	0.134	0.295
isthmuscingulate	1.610	-0.105	-0.003	0.032	0.108	0.295
lateraloccipital	1.286	-0.089	-0.006	0.028	0.199	0.355
lateralorbitofrontal	-0.553	0.053	-0.020	0.011	0.580	0.759
lingual	-1.340	0.111	-0.028	0.005	0.180	0.341
medialorbitofrontal	1.033	-0.061	-0.007	0.024	0.302	0.479
middletemporal	-5.247	0.394	-0.067	-0.031	**1.60E-07**	**5.45E-06**
parahippocampal	1.549	-0.106	-0.006	0.051	0.121	0.295
paracentral	-1.015	0.089	-0.027	0.009	0.310	0.479
parsopercularis	0.328	-0.017	-0.013	0.018	0.743	0.796
parsorbitalis	-3.990	0.303	-0.061	-0.021	**6.69E-05**	**1.14E-03**
parstriangularis	-3.442	0.265	-0.044	-0.012	**0.001**	**0.007**
pericalcarine	1.226	-0.085	-0.008	0.033	0.220	0.374
postcentral	-0.790	0.069	-0.025	0.011	0.429	0.635
posteriorcingulate	0.290	-0.009	-0.012	0.017	0.772	0.796
precentral	-2.295	0.171	-0.037	-0.003	**0.022**	0.074
precuneus	0.463	-0.014	-0.011	0.017	0.644	0.782
rostralanteriorcingulate	-0.399	0.038	-0.026	0.017	0.690	0.782
rostralmiddlefrontal	-3.203	0.250	-0.038	-0.009	**0.001**	**0.012**
superiorfrontal	-2.509	0.196	-0.037	-0.005	**0.012**	0.052
superiorparietal	-0.296	0.037	-0.017	0.013	0.767	0.796
superiortemporal	0.624	-0.040	-0.012	0.024	0.533	0.725
supramarginal	-0.459	0.046	-0.020	0.012	0.646	0.782
frontalpole	-1.340	0.108	-0.048	0.009	0.180	0.341
temporalpole	-1.573	0.120	-0.060	0.007	0.116	0.295
transversetemporal	-0.404	0.036	-0.027	0.018	0.686	0.782
insula	-2.818	0.212	-0.045	-0.008	**0.005**	**0.033**

Table S2h: Average dysmaturation outcomes of surface area in the ABCD-12 cohort

ROI	T_{5073}	Cohen's d	95% CI lower	95% CI upper	p-value	p_{FDR}
bankssts	-2.462	0.204	-54.377	-6.170	**0.014**	**0.047**
caudalanteriorcingulate	-0.364	0.033	-24.862	17.073	0.716	0.811
caudalmiddlefrontal	-1.559	0.131	-111.718	12.735	0.119	0.212
cuneus	-0.689	0.060	-45.986	22.075	0.491	0.596
entorhinal	1.482	-0.114	-3.299	23.757	0.138	0.224
fusiform	-2.303	0.181	-122.981	-9.894	**0.021**	0.066
inferiorparietal	-3.341	0.269	-306.606	-79.852	**0.001**	**0.007**
inferiortemporal	0.121	-0.002	-66.141	74.838	0.904	0.931
isthmuscingulate	-2.707	0.218	-54.526	-8.725	**0.007**	**0.038**
lateraloccipital	-1.841	0.149	-195.042	6.110	0.066	0.131
lateralorbitofrontal	-1.957	0.161	-101.786	0.100	0.050	0.107
lingual	-0.733	0.064	-92.102	41.992	0.464	0.584
medialorbitofrontal	-1.311	0.109	-58.563	11.635	0.190	0.269
middletemporal	-2.657	0.214	-167.325	-25.248	**0.008**	**0.038**
parahippocampal	-1.550	0.126	-22.714	2.656	0.121	0.212
paracentral	0.087	0.001	-26.881	29.370	0.931	0.931
parsopercularis	-2.553	0.213	-89.179	-11.707	**0.011**	**0.040**
parsorbitalis	-0.250	0.026	-18.794	14.549	0.803	0.881
parstriangularis	-3.665	0.294	-105.567	-31.985	**2.50E-04**	**0.007**
pericalcarine	0.651	-0.051	-27.051	53.953	0.515	0.604
postcentral	-2.610	0.214	-183.014	-26.014	**0.009**	**0.039**
posteriorcingulate	-2.061	0.169	-55.831	-1.390	**0.039**	0.096
precentral	-1.355	0.113	-143.226	26.170	0.176	0.260
precuneus	-1.536	0.128	-147.572	17.900	0.125	0.212
rostralanteriorcingulate	-2.245	0.184	-44.299	-2.993	**0.025**	0.070
rostralmiddlefrontal	0.185	-0.006	-113.360	136.969	0.853	0.907
superiorfrontal	-1.416	0.115	-259.843	41.872	0.157	0.242
superiorparietal	-3.220	0.268	-313.824	-76.314	**0.001**	**0.009**
superiortemporal	-2.021	0.164	-147.503	-2.248	**0.043**	0.098
supramarginal	-1.175	0.101	-158.680	39.723	0.240	0.326
frontalpole	0.761	-0.051	-3.846	8.725	0.447	0.584
temporalpole	3.516	-0.269	7.679	27.035	**4.42E-04**	**0.007**
transversetemporal	-2.203	0.182	-18.769	-1.094	**0.028**	0.072
insula	-3.377	0.264	-107.629	-28.559	**0.001**	**0.007**

Table S2i: Average dysmaturation outcomes of cerebral tissue volume measures in the ABCD-12 cohort

ROI	T_{5949}	Cohen's d	95% CI lower	95% CI upper	p-value	p_{FDR}
WMV	-1.473	0.085	-11392.596	1618.368	0.141	0.141
GMV	-3.713	0.243	-24275.403	-7499.099	**2.07e-04**	**8.27e-04**
sGMV	-3.233	0.207	-1712.964	-419.774	**0.001**	**0.002**
mean CTh	-1.799	0.147	-0.022	0.001	0.072	0.096

Table S2j: Average dysmaturation outcomes of cortical thickness in the BLS-26 cohort

ROI	T_{199}	Cohen's d	95% CI lower	95% CI upper	p-value	p_{FDR}
bankssts	-2.969	0.428	-0.086	-0.017	**0.003**	**0.013**
caudalanteriorcingulate	1.010	-0.135	-0.021	0.064	0.314	0.431
caudalmiddlefrontal	-1.041	0.155	-0.054	0.017	0.299	0.431
cuneus	-0.565	0.094	-0.040	0.022	0.572	0.671
entorhinal	-1.347	0.194	-0.104	0.020	0.179	0.305
fusiform	-2.666	0.367	-0.081	-0.012	**0.008**	**0.022**
inferiorparietal	-4.632	0.665	-0.103	-0.041	**6.53E-06**	**7.40E-05**
inferiortemporal	-1.746	0.259	-0.057	0.003	0.082	0.165
isthmuscingulate	3.071	-0.416	0.022	0.103	**0.002**	**0.012**
lateraloccipital	-0.054	0.033	-0.030	0.029	0.957	0.975
lateralorbitofrontal	-0.458	0.076	-0.036	0.023	0.648	0.710
lingual	-1.220	0.184	-0.052	0.012	0.224	0.363
medialorbitofrontal	3.398	-0.466	0.023	0.087	**0.001**	**0.005**
middletemporal	-6.518	0.929	-0.146	-0.078	**5.72E-10**	**1.94E-08**
parahippocampal	-0.478	0.082	-0.066	0.040	0.633	0.710
paracentral	1.630	-0.241	-0.007	0.074	0.105	0.198
parsopercularis	-1.900	0.275	-0.058	0.001	0.059	0.125
parsorbitalis	-6.108	0.868	-0.173	-0.088	**5.22E-09**	**8.87E-08**
parstriangularis	-2.909	0.415	-0.079	-0.015	**0.004**	**0.014**
pericalcarine	0.877	-0.119	-0.017	0.045	0.381	0.499
postcentral	-2.251	0.312	-0.068	-0.004	**0.026**	0.058
posteriorcingulate	2.393	-0.291	0.007	0.068	**0.018**	**0.043**
precentral	-2.752	0.395	-0.110	-0.018	**0.006**	**0.018**
precuneus	-1.003	0.152	-0.050	0.016	0.317	0.431
rostralanteriorcingulate	0.823	-0.111	-0.025	0.061	0.411	0.517
rostralmiddlefrontal	-3.565	0.514	-0.078	-0.022	**4.55E-04**	**0.003**
superiorfrontal	0.799	-0.111	-0.019	0.045	0.425	0.517
superiorparietal	-2.858	0.409	-0.076	-0.014	**0.005**	**0.015**
superiortemporal	-1.029	0.152	-0.055	0.017	0.305	0.431
supramarginal	-3.749	0.534	-0.089	-0.028	**2.33E-04**	**0.002**
frontalpole	-0.305	0.053	-0.063	0.046	0.760	0.808
temporalpole	-1.443	0.195	-0.122	0.019	0.151	0.270
transversetemporal	0.031	-0.003	-0.052	0.054	0.975	0.975
insula	-2.969	0.422	-0.075	-0.015	**0.003**	**0.013**

Table S2k: Average dysmaturation outcomes of surface area in the BLS-26 cohort

ROI	T_{199}	Cohen's d	95% CI lower	95% CI upper	p-value	p_{FDR}
bankssts	-4.382	0.597	-109.629	-41.585	**1.90E-05**	**1.29E-04**
caudalanteriorcingulate	-2.670	0.371	-70.799	-10.653	**0.008**	**0.025**
caudalmiddlefrontal	-0.778	0.131	-115.861	50.326	0.438	0.496
cuneus	-2.431	0.331	-125.404	-13.062	**0.016**	**0.042**
entorhinal	2.062	-0.259	1.082	48.530	**0.041**	0.073
fusiform	-2.740	0.346	-202.674	-33.043	**0.007**	**0.025**
inferiorparietal	-3.058	0.396	-450.857	-97.334	**0.003**	**0.011**
inferiortemporal	-0.143	0.050	-117.263	101.398	0.886	0.913
isthmuscingulate	-5.889	0.770	-136.447	-67.993	**1.63E-08**	**5.54E-07**
lateraloccipital	-0.520	0.088	-198.815	115.801	0.603	0.662
lateralorbitofrontal	-4.807	0.635	-256.512	-107.267	**3.02E-06**	**5.14E-05**
lingual	-2.264	0.317	-241.986	-16.690	**0.025**	0.052
medialorbitofrontal	-2.670	0.359	-118.432	-17.815	**0.008**	**0.025**
middletemporal	-4.601	0.583	-333.062	-133.213	**7.48E-06**	**8.48E-05**
parahippocampal	-1.140	0.163	-32.193	8.603	0.256	0.323
paracentral	-1.971	0.271	-86.325	0.025	0.050	0.081
parsopercularis	-0.458	0.072	-73.972	46.106	0.648	0.688
parsorbitalis	-1.991	0.278	-51.575	-0.247	**0.048**	0.081
parstriangularis	-2.138	0.296	-117.332	-4.742	**0.034**	0.064
pericalcarine	-1.942	0.272	-136.885	1.046	0.054	0.083
postcentral	-1.897	0.245	-202.921	3.933	0.059	0.088
posteriorcingulate	-4.449	0.586	-140.524	-54.209	**1.43E-05**	**1.22E-04**
precentral	-2.217	0.289	-269.980	-15.783	**0.028**	0.056
precuneus	-1.138	0.165	-168.776	45.227	0.256	0.323
rostralanteriorcingulate	-3.971	0.528	-93.691	-31.519	**9.98E-05**	**5.66E-04**
rostralmiddlefrontal	-0.034	0.037	-185.397	179.144	0.973	0.973
superiorfrontal	-2.375	0.300	-422.883	-39.184	**0.019**	**0.044**
superiorparietal	-3.287	0.429	-395.185	-98.796	**0.001**	**0.006**
superiortemporal	-0.857	0.137	-158.440	62.482	0.393	0.477
supramarginal	-2.353	0.316	-315.498	-27.769	**0.020**	**0.044**
frontalpole	0.829	-0.086	-4.526	11.098	0.408	0.478
temporalpole	1.348	-0.137	-5.357	28.494	0.179	0.244
transversetemporal	-2.618	0.369	-34.076	-4.795	**0.010**	**0.027**
insula	-1.764	0.245	-115.719	6.429	0.079	0.112

Table S2l: Average dysmaturation outcomes of cerebral tissue volume measures in the BLS-26 cohort

**ROI**	**T_{199}**	**Cohen's d**	**95% CI lower**	**95% CI upper**	**p-value**	**p_{FDR}**
WMV	-4.686	0.589	-47394.724	-19318.009	**5.17E-06**	**6.89E-06**
GMV	-5.298	0.586	-46750.234	-21388.966	**3.09E-07**	**6.18E-07**
sGMV	-9.216	1.134	-7390.032	-4784.843	**4.48E-17**	**1.79E-16**
mean CTh	-2.423	0.353	-0.052	-0.005	**0.016**	**0.016**

Table S2m: Average dysmaturation outcomes of cortical thickness in the BLS-38 cohort

ROI	T_{101}	Cohen's d	95% CI lower	95% CI upper	p-value	p_{FDR}
bankssts	-1.884	0.338	-0.083	0.002	0.062	0.262
caudalanteriorcingulate	1.168	-0.263	-0.025	0.098	0.246	0.514
caudalmiddlefrontal	-0.429	0.057	-0.051	0.033	0.669	0.700
cuneus	-0.842	0.166	-0.059	0.024	0.402	0.571
entorhinal	-0.500	0.034	-0.129	0.077	0.618	0.678
fusiform	-1.675	0.264	-0.070	0.006	0.097	0.302
inferiorparietal	-3.189	0.621	-0.089	-0.021	**0.002**	0.065
inferiortemporal	1.012	-0.251	-0.020	0.061	0.314	0.514
isthmuscingulate	1.076	-0.249	-0.026	0.087	0.285	0.514
lateraloccipital	-0.651	0.146	-0.050	0.025	0.516	0.627
lateralorbitofrontal	1.523	-0.355	-0.010	0.077	0.131	0.342
lingual	-0.840	0.152	-0.059	0.024	0.403	0.571
medialorbitofrontal	2.450	-0.515	0.011	0.105	**0.016**	0.136
middletemporal	-2.926	0.544	-0.112	-0.022	**0.004**	0.072
parahippocampal	-1.211	0.227	-0.134	0.032	0.229	0.514
paracentral	0.414	-0.096	-0.041	0.063	0.680	0.700
parsopercularis	-0.670	0.090	-0.055	0.027	0.504	0.627
parsorbitalis	-2.682	0.512	-0.131	-0.020	**0.009**	0.097
parstriangularis	-1.139	0.212	-0.067	0.018	0.257	0.514
pericalcarine	-0.565	0.149	-0.053	0.029	0.573	0.650
postcentral	-1.672	0.293	-0.070	0.006	0.098	0.302
posteriorcingulate	0.272	-0.092	-0.033	0.044	0.786	0.786
precentral	-1.079	0.194	-0.070	0.021	0.283	0.514
precuneus	-0.594	0.089	-0.050	0.027	0.554	0.649
rostralanteriorcingulate	1.054	-0.234	-0.035	0.113	0.294	0.514
rostralmiddlefrontal	-1.853	0.312	-0.067	0.002	0.067	0.262
superiorfrontal	0.742	-0.177	-0.028	0.060	0.460	0.601
superiorparietal	-2.010	0.384	-0.093	-0.001	**0.047**	0.262
superiortemporal	0.802	-0.201	-0.027	0.064	0.424	0.577
supramarginal	-1.563	0.259	-0.067	0.008	0.121	0.342
frontalpole	2.012	-0.437	0.001	0.129	**0.047**	0.262
temporalpole	-1.005	0.168	-0.124	0.041	0.317	0.514
transversetemporal	-0.908	0.141	-0.098	0.037	0.366	0.566
insula	-1.835	0.329	-0.095	0.004	0.069	0.262

Table S2n: Average dysmaturation outcomes of surface area in the BLS-38 cohort

ROI	T_{101}	Cohen's d	95% CI lower	95% CI upper	p-value	p_{FDR}
bankssts	-4.559	0.808	-137.029	-53.934	**1.45E-05**	**2.46E-04**
caudalanteriorcingulate	-0.903	0.128	-62.132	23.271	0.369	0.464
caudalmiddlefrontal	0.348	-0.049	-74.797	106.620	0.729	0.799
cuneus	-1.107	0.178	-110.665	31.404	0.271	0.384
entorhinal	-0.085	0.034	-31.904	29.279	0.932	0.932
fusiform	-2.660	0.407	-261.026	-38.036	**0.009**	**0.044**
inferiorparietal	-2.966	0.490	-562.834	-111.735	**0.004**	**0.032**
inferiortemporal	-1.497	0.224	-260.985	36.500	0.138	0.209
isthmuscingulate	-2.245	0.377	-98.823	-6.110	**0.027**	0.070
lateraloccipital	-0.213	0.029	-226.662	182.750	0.832	0.884
lateralorbitofrontal	-3.785	0.649	-301.073	-93.997	**2.61E-04**	**0.003**
lingual	-1.796	0.333	-289.557	14.354	0.075	0.142
medialorbitofrontal	-2.379	0.331	-136.582	-12.371	**0.019**	0.061
middletemporal	-4.712	0.762	-443.340	-180.631	**7.87E-06**	**2.46E-04**
parahippocampal	-2.310	0.380	-55.672	-4.230	**0.023**	0.065
paracentral	-2.218	0.410	-124.502	-6.947	**0.029**	0.070
parsopercularis	-0.784	0.137	-107.049	46.414	0.435	0.513
parsorbitalis	-1.953	0.346	-66.159	0.517	0.054	0.114
parstriangularis	-2.026	0.358	-147.828	-1.553	**0.045**	0.103
pericalcarine	-0.465	0.083	-115.380	71.585	0.643	0.729
postcentral	-1.839	0.296	-246.847	9.353	0.069	0.138
posteriorcingulate	-1.614	0.263	-101.786	10.467	0.110	0.186
precentral	-1.710	0.296	-246.444	18.230	0.090	0.162
precuneus	-1.039	0.157	-248.639	77.704	0.301	0.410
rostralanteriorcingulate	-1.481	0.231	-75.568	10.964	0.142	0.209
rostralmiddlefrontal	-0.971	0.143	-334.446	114.580	0.334	0.436
superiorfrontal	-2.368	0.383	-538.259	-47.481	**0.020**	0.061
superiorparietal	-2.763	0.456	-456.018	-74.893	**0.007**	**0.040**
superiortemporal	-0.779	0.109	-198.875	86.733	0.438	0.513
supramarginal	-2.748	0.448	-460.860	-74.424	**0.007**	**0.040**
frontalpole	0.180	-0.056	-9.294	11.148	0.858	0.884
temporalpole	2.393	-0.451	4.407	47.163	**0.019**	0.061
transversetemporal	-2.372	0.445	-49.968	-4.451	**0.020**	0.061
insula	-1.548	0.244	-155.240	19.133	0.125	0.202

Table S2o: Average dysmaturation outcomes of cerebral tissue volume measures in the BLS-38 cohort

**ROI**	**T_{101}**	**Cohen's d**	**95% CI lower**	**95% CI upper**	**p-value**	**p_{FDR}**
WMV	-4.221	0.610	-56845.828	-20499.850	**5.32E-05**	**1.06E-04**
GMV	-4.075	0.565	-47724.968	-16471.827	**9.18E-05**	**1.22E-04**
sGMV	-7.238	1.149	-9396.397	-5353.749	**9.12E-11**	**3.65E-10**
mean CTh	-1.264	0.199	-0.042	0.009	0.209	0.209

Tables S3: Comparison between the Bethlehem-framework and the Rutherford-framework

See Supplementary Table S1 for region abbreviations. Two-sided p-values (uncorrected as well as corrected for the False Discovery Rate, FDR) are provided in the following.

Table S3a: Spearman correlation coefficients between cortical thickness deviation score estimates based on the Bethlehem-framework and the Rutherford-framework

ROI	Spearman_rho(94)	95% CI lower	95% CI upper	p-value	p_{FDR}
bankssts	0.990	0.984	0.993	**7.50E-81**	**3.19E-80**
caudalanteriorcingulate	0.918	0.880	0.945	**1.30E-39**	**1.33E-39**
caudalmiddlefrontal	0.972	0.958	0.981	**6.78E-61**	**9.22E-61**
cuneus	0.981	0.971	0.987	**2.49E-68**	**5.65E-68**
entorhinal	0.991	0.986	0.994	**2.11E-83**	**1.02E-82**
fusiform	0.991	0.986	0.994	**7.96E-84**	**4.51E-83**
inferiorparietal	0.979	0.968	0.986	**2.10E-66**	**4.20E-66**
inferiortemporal	0.987	0.981	0.991	**1.30E-76**	**4.41E-76**
isthmuscingulate	0.881	0.826	0.919	**2.79E-32**	**2.79E-32**
lateraloccipital	0.986	0.980	0.991	**1.42E-75**	**4.40E-75**
lateralorbitofrontal	0.995	0.993	0.997	**7.21E-98**	**2.45E-96**
lingual	0.970	0.955	0.980	**2.38E-59**	**3.11E-59**
medialorbitofrontal	0.978	0.967	0.985	**6.38E-66**	**1.20E-65**
middletemporal	0.973	0.960	0.982	**9.90E-62**	**1.46E-61**
parahippocampal	0.927	0.892	0.951	**7.73E-42**	**8.21E-42**
paracentral	0.974	0.961	0.983	**2.11E-62**	**3.26E-62**
parsopercularis	0.982	0.973	0.988	**1.13E-69**	**2.94E-69**
parsorbitalis	0.964	0.947	0.976	**5.70E-56**	**7.18E-56**
parstriangularis	0.993	0.990	0.995	**9.77E-90**	**8.30E-89**
pericalcarine	0.959	0.939	0.972	**3.32E-53**	**3.76E-53**
postcentral	0.976	0.964	0.984	**7.30E-64**	**1.18E-63**
posteriorcingulate	0.934	0.902	0.955	**1.02E-43**	**1.12E-43**
precentral	0.979	0.969	0.986	**1.12E-66**	**2.39E-66**
precuneus	0.994	0.990	0.996	**1.07E-90**	**1.21E-89**
rostralanteriorcingulate	0.961	0.942	0.974	**3.76E-54**	**4.41E-54**
rostralmiddlefrontal	0.993	0.989	0.995	**9.40E-88**	**6.39E-87**
superiorfrontal	0.963	0.944	0.975	**4.08E-55**	**4.95E-55**
superiorparietal	0.978	0.967	0.985	**9.27E-66**	**1.66E-65**
superiortemporal	0.995	0.992	0.997	**1.45E-95**	**2.47E-94**
supramarginal	0.977	0.966	0.985	**8.38E-65**	**1.42E-64**
frontalpole	0.981	0.971	0.987	**1.25E-68**	**3.04E-68**
temporalpole	0.989	0.983	0.993	**1.59E-79**	**5.99E-79**

transversetemporal	0.986	0.980	0.991	**2.16E-75**	**6.12E-75**
insula	0.972	0.959	0.982	**3.29E-61**	**4.66E-61**

Table S3b: Spearman correlation coefficients between surface area deviation score estimates based on the Bethlehem-framework and the Rutherford-framework

**ROI**	**Spearman_rho(94)**	**95% CI lower**	**95% CI upper**	**p-value**	**p_{FDR}**
bankssts	0.826	0.749	0.880	**4.24E-25**	**1.75E-24**
caudalanteriorcingulate	0.879	0.823	0.918	**5.93E-32**	**9.78E-31**
caudalmiddlefrontal	0.762	0.662	0.835	**2.13E-19**	**3.69E-19**
cuneus	0.768	0.671	0.839	**7.01E-20**	**1.40E-19**
fusiform	0.663	0.533	0.762	**1.85E-13**	**1.91E-13**
inferiorparietal	0.789	0.699	0.854	**1.37E-21**	**3.23E-21**
inferiortemporal	0.660	0.530	0.760	**2.52E-13**	**2.52E-13**
isthmuscingulate	0.705	0.588	0.794	**1.04E-15**	**1.38E-15**
lateraloccipital	0.720	0.608	0.805	**1.31E-16**	**1.88E-16**
lateralorbitofrontal	0.712	0.596	0.798	**4.40E-16**	**6.05E-16**
lingual	0.816	0.735	0.873	**4.63E-24**	**1.53E-23**
medialorbitofrontal	0.673	0.546	0.770	**5.90E-14**	**6.28E-14**
middletemporal	0.678	0.553	0.774	**3.18E-14**	**3.50E-14**
parahippocampal	0.840	0.769	0.891	**1.05E-26**	**5.80E-26**
paracentral	0.833	0.760	0.886	**6.18E-26**	**2.91E-25**
parsopercularis	0.850	0.783	0.897	**6.92E-28**	**5.71E-27**
parsorbitalis	0.768	0.670	0.839	**7.20E-20**	**1.40E-19**
parstriangularis	0.823	0.745	0.878	**8.55E-25**	**3.13E-24**
pericalcarine	0.886	0.833	0.922	**4.70E-33**	**1.55E-31**
postcentral	0.763	0.665	0.836	**1.52E-19**	**2.79E-19**
posteriorcingulate	0.840	0.769	0.891	**1.02E-26**	**5.80E-26**
precentral	0.687	0.564	0.780	**1.08E-14**	**1.23E-14**
precuneus	0.751	0.649	0.827	**1.16E-18**	**1.82E-18**
rostralanteriorcingulate	0.799	0.713	0.862	**1.72E-22**	**4.36E-22**
rostralmiddlefrontal	0.755	0.654	0.830	**6.04E-19**	**9.96E-19**
superiorfrontal	0.690	0.568	0.782	**7.41E-15**	**9.06E-15**
superiorparietal	0.811	0.729	0.870	**1.27E-23**	**3.48E-23**
superiortemporal	0.691	0.569	0.783	**6.85E-15**	**8.70E-15**
supramarginal	0.777	0.684	0.846	**1.22E-20**	**2.68E-20**
frontalpole	0.751	0.647	0.827	**1.34E-18**	**2.01E-18**
temporalpole	0.813	0.732	0.872	**8.01E-24**	**2.40E-23**
transversetemporal	0.861	0.798	0.905	**2.61E-29**	**2.87E-28**
insula	0.689	0.566	0.781	**9.04E-15**	**1.06E-14**

Supplementary Tables S4: Intraclass correlation coefficients (ICC) for longitudinal IBAP consistency within individuals

See Supplementary Table S1 for region abbreviations. Intraclass Correlation Coefficients (ICC) were calculated between the two timepoints across subjects. Two-sided p-values (uncorrected as well as corrected for the False Discovery Rate, FDR) for these correlations are provided in the following.

Table S4a: Intraclass correlation coefficients of surface area in the ABCD cohort

ROI	ICC_{147}	95% CI lower	95% CI upper	p-value	p_{FDR}
bankssts	0.951	0.93	0.96	**3.90E-77**	**9.20E-77**
caudalanteriorcingulate	0.943	0.92	0.96	**2.03E-72**	**3.46E-72**
caudalmiddlefrontal	0.969	0.96	0.98	**2.52E-91**	**2.08E-90**
cuneus	0.962	0.95	0.97	**4.64E-85**	**1.70E-84**
fusiform	0.958	0.94	0.97	**4.45E-82**	**1.34E-81**
inferiorparietal	0.966	0.95	0.98	**1.54E-88**	**7.25E-88**
inferiortemporal	0.964	0.95	0.97	**4.38E-87**	**1.81E-86**
isthmuscingulate	0.945	0.92	0.96	**1.41E-73**	**3.11E-73**
lateraloccipital	0.969	0.96	0.98	**1.60E-91**	**1.76E-90**
lateralorbitofrontal	0.866	0.82	0.90	**2.44E-46**	**2.78E-46**
lingual	0.970	0.96	0.98	**1.90E-92**	**3.13E-91**
medialorbitofrontal	0.860	0.81	0.90	**5.16E-45**	**5.68E-45**
middletemporal	0.957	0.94	0.97	**5.82E-81**	**1.60E-80**
parahippocampal	0.882	0.84	0.91	**3.22E-50**	**3.79E-50**
paracentral	0.914	0.88	0.94	**1.26E-59**	**1.74E-59**
parsopercularis	0.943	0.92	0.96	**1.46E-72**	**2.68E-72**
parsorbitalis	0.926	0.90	0.95	**3.03E-64**	**4.77E-64**
parstriangularis	0.945	0.92	0.96	**2.15E-73**	**4.18E-73**
pericalcarine	0.953	0.94	0.97	**2.57E-78**	**6.52E-78**
postcentral	0.914	0.88	0.94	**1.34E-59**	**1.77E-59**
posteriorcingulate	0.967	0.96	0.98	**8.74E-90**	**5.77E-89**
precentral	0.912	0.88	0.94	**3.92E-59**	**4.97E-59**
precuneus	0.967	0.95	0.98	**4.00E-89**	**2.20E-88**
rostralanteriorcingulate	0.893	0.85	0.92	**5.04E-53**	**6.16E-53**
rostralmiddlefrontal	0.916	0.89	0.94	**2.00E-60**	**2.87E-60**
superiorfrontal	0.972	0.96	0.98	**1.07E-94**	**3.53E-93**
superiorparietal	0.945	0.92	0.96	**1.67E-73**	**3.44E-73**
superiortemporal	0.959	0.94	0.97	**1.89E-82**	**6.24E-82**
supramarginal	0.943	0.92	0.96	**2.10E-72**	**3.46E-72**
frontalpole	0.784	0.71	0.84	**1.69E-32**	**1.69E-32**
temporalpole	0.785	0.71	0.84	**1.07E-32**	**1.11E-32**

transversetemporal	0.925	0.90	0.95	**4.85E-64**	**7.27E-64**
insula	0.837	0.78	0.88	**1.36E-40**	**1.45E-40**

Table S4b: Intraclass correlation coefficients of surface area in the BLS cohort

ROI	ICC_{45}	95% CI lower	95% CI upper	p-value	p_{FDR}
bankssts	0.883	0.80	0.93	**1.10E-16**	**1.34E-16**
caudalanteriorcingulate	0.950	0.91	0.97	**1.08E-24**	**3.96E-24**
caudalmiddlefrontal	0.939	0.89	0.97	**9.04E-23**	**2.13E-22**
cuneus	0.944	0.90	0.97	**1.26E-23**	**3.78E-23**
fusiform	0.905	0.84	0.95	**1.21E-18**	**1.81E-18**
inferiorparietal	0.968	0.94	0.98	**5.87E-29**	**6.46E-28**
inferiortemporal	0.906	0.84	0.95	**1.02E-18**	**1.60E-18**
isthmuscingulate	0.887	0.81	0.94	**4.82E-17**	**6.37E-17**
lateraloccipital	0.942	0.90	0.97	**2.88E-23**	**7.92E-23**
lateralorbitofrontal	0.875	0.78	0.93	**4.77E-16**	**5.62E-16**
lingual	0.950	0.91	0.97	**9.48E-25**	**3.96E-24**
medialorbitofrontal	0.836	0.72	0.91	**1.30E-13**	**1.43E-13**
middletemporal	0.901	0.83	0.94	**3.39E-18**	**4.67E-18**
parahippocampal	0.913	0.85	0.95	**1.79E-19**	**2.95E-19**
paracentral	0.938	0.89	0.97	**1.32E-22**	**2.90E-22**
parsopercularis	0.967	0.94	0.98	**1.11E-28**	**9.14E-28**
parsorbitalis	0.924	0.87	0.96	**9.72E-21**	**1.78E-20**
parstriangularis	0.950	0.91	0.97	**9.81E-25**	**3.96E-24**
pericalcarine	0.948	0.91	0.97	**2.69E-24**	**8.88E-24**
postcentral	0.936	0.89	0.96	**2.53E-22**	**5.22E-22**
posteriorcingulate	0.940	0.89	0.97	**5.54E-23**	**1.41E-22**
precentral	0.883	0.80	0.93	**1.07E-16**	**1.34E-16**
precuneus	0.983	0.97	0.99	**6.32E-35**	**1.04E-33**
rostralanteriorcingulate	0.869	0.77	0.93	**1.27E-15**	**1.45E-15**
rostralmiddlefrontal	0.919	0.86	0.95	**4.47E-20**	**7.77E-20**
superiorfrontal	0.966	0.94	0.98	**2.00E-28**	**1.32E-27**
superiorparietal	0.989	0.98	0.99	**2.10E-39**	**6.93E-38**
superiortemporal	0.930	0.88	0.96	**1.82E-21**	**3.54E-21**
supramarginal	0.953	0.92	0.97	**2.62E-25**	**1.44E-24**
frontalpole	0.664	0.47	0.80	**1.82E-07**	**1.87E-07**
temporalpole	0.647	0.44	0.79	**4.56E-07**	**4.56E-07**
transversetemporal	0.904	0.83	0.95	**1.68E-18**	**2.40E-18**
insula	0.803	0.67	0.89	**5.74E-12**	**6.11E-12**

Table S4c: Intraclass correlation coefficients of cortical thickness in the ABCD cohort

ROI	ICC_{190}	95% CI lower	95% CI upper	p-value	p_{FDR}
bankssts	0.874	0.84	0.90	**1.00E-61**	**1.71E-60**
caudalanteriorcingulate	0.808	0.75	0.85	**7.40E-46**	**5.03E-45**
caudalmiddlefrontal	0.735	0.66	0.79	**3.10E-34**	**4.79E-34**
cuneus	0.798	0.74	0.84	**5.77E-44**	**2.80E-43**
entorhinal	0.633	0.54	0.71	**3.58E-23**	**4.20E-23**
fusiform	0.748	0.68	0.80	**5.51E-36**	**8.93E-36**
inferiorparietal	0.779	0.72	0.83	**1.32E-40**	**3.75E-40**
inferiortemporal	0.749	0.68	0.81	**4.48E-36**	**8.12E-36**
isthmuscingulate	0.876	0.84	0.91	**1.61E-62**	**5.47E-61**
lateraloccipital	0.801	0.74	0.85	**1.63E-44**	**9.26E-44**
lateralorbitofrontal	0.606	0.51	0.69	**6.26E-21**	**7.10E-21**
lingual	0.784	0.72	0.83	**1.98E-41**	**7.48E-41**
medialorbitofrontal	0.605	0.51	0.69	**7.94E-21**	**8.70E-21**
middletemporal	0.782	0.72	0.83	**3.93E-41**	**1.21E-40**
parahippocampal	0.830	0.78	0.87	**1.85E-50**	**2.10E-49**
paracentral	0.774	0.71	0.83	**7.31E-40**	**1.91E-39**
parsopercularis	0.769	0.70	0.82	**4.41E-39**	**1.00E-38**
parsorbitalis	0.692	0.61	0.76	**5.33E-29**	**7.25E-29**
parstriangularis	0.770	0.70	0.82	**3.64E-39**	**8.83E-39**
pericalcarine	0.749	0.68	0.81	**4.54E-36**	**8.12E-36**
postcentral	0.794	0.74	0.84	**2.74E-43**	**1.16E-42**
posteriorcingulate	0.810	0.76	0.85	**2.73E-46**	**2.32E-45**
precentral	0.705	0.63	0.77	**2.06E-30**	**2.91E-30**
precuneus	0.782	0.72	0.83	**3.33E-41**	**1.13E-40**
rostralanteriorcingulate	0.582	0.48	0.67	**4.15E-19**	**4.41E-19**
rostralmiddlefrontal	0.661	0.57	0.73	**8.87E-26**	**1.08E-25**
superiorfrontal	0.748	0.68	0.80	**4.99E-36**	**8.49E-36**
superiorparietal	0.731	0.66	0.79	**1.10E-33**	**1.63E-33**
superiortemporal	0.767	0.70	0.82	**7.97E-39**	**1.69E-38**
supramarginal	0.753	0.68	0.81	**1.11E-36**	**2.23E-36**
frontalpole	0.689	0.61	0.76	**1.29E-28**	**1.68E-28**
temporalpole	0.444	0.32	0.55	**5.40E-11**	**5.56E-11**
transversetemporal	0.663	0.58	0.74	**5.96E-26**	**7.51E-26**
insula	0.394	0.27	0.51	**8.00E-09**	**8.00E-09**

Table S4d: Intraclass correlation coefficients of cortical thickness in the BLS cohort

**ROI**	**ICC_{45}**	**95% CI lower**	**95% CI upper**	**p-value**	**p_{FDR}**
bankssts	0.795	0.66	0.88	**1.25E-11**	**5.30E-11**
caudalanteriorcingulate	0.736	0.57	0.84	**1.87E-09**	**4.89E-09**
caudalmiddlefrontal	0.705	0.52	0.83	**1.55E-08**	**3.09E-08**
cuneus	0.775	0.63	0.87	**8.22E-11**	**2.79E-10**
entorhinal	0.484	0.23	0.68	**2.81E-04**	**2.98E-04**
fusiform	0.426	0.16	0.64	**1.40E-03**	**1.44E-03**
inferiorparietal	0.797	0.66	0.88	**1.04E-11**	**5.05E-11**
inferiortemporal	0.619	0.40	0.77	**1.79E-06**	**2.76E-06**
isthmuscingulate	0.558	0.32	0.73	**2.29E-05**	**3.00E-05**
lateraloccipital	0.638	0.43	0.78	**6.91E-07**	**1.12E-06**
lateralorbitofrontal	0.539	0.30	0.72	**4.59E-05**	**5.38E-05**
lingual	0.561	0.33	0.73	**2.05E-05**	**2.79E-05**
medialorbitofrontal	0.538	0.30	0.71	**4.80E-05**	**5.44E-05**
middletemporal	0.591	0.37	0.75	**6.21E-06**	**9.18E-06**
parahippocampal	0.759	0.60	0.86	**3.22E-10**	**9.13E-10**
paracentral	0.804	0.67	0.89	**5.08E-12**	**3.46E-11**
parsopercularis	0.766	0.61	0.86	**1.74E-10**	**5.38E-10**
parsorbitalis	0.808	0.68	0.89	**3.33E-12**	**2.83E-11**
parstriangularis	0.667	0.47	0.80	**1.52E-07**	**2.58E-07**
pericalcarine	0.520	0.27	0.70	**9.12E-05**	**1.00E-04**
postcentral	0.813	0.69	0.89	**2.02E-12**	**2.83E-11**
posteriorcingulate	0.687	0.50	0.81	**4.93E-08**	**9.32E-08**
precentral	0.672	0.48	0.80	**1.14E-07**	**2.05E-07**
precuneus	0.810	0.68	0.89	**2.57E-12**	**2.83E-11**
rostralanteriorcingulate	0.719	0.54	0.83	**6.43E-09**	**1.46E-08**
rostralmiddlefrontal	0.568	0.33	0.74	**1.58E-05**	**2.24E-05**
superiorfrontal	0.779	0.63	0.87	**5.61E-11**	**2.12E-10**
superiorparietal	0.799	0.66	0.88	**8.41E-12**	**4.76E-11**
superiortemporal	0.709	0.53	0.83	**1.25E-08**	**2.66E-08**
supramarginal	0.844	0.73	0.91	**4.62E-14**	**1.57E-12**
frontalpole	0.548	0.31	0.72	**3.30E-05**	**4.16E-05**
temporalpole	-0.093	-0.37	0.20	0.733	0.733
transversetemporal	0.720	0.54	0.83	**6.01E-09**	**1.46E-08**
insula	0.545	0.31	0.72	**3.68E-05**	**4.47E-05**

Supplementary Table S5: Association between strength of the spatial relationship between cellular distributions and cortical thickness deviation profiles with gestational age

**Cell type**	**Spearman rho(94)**	**95 % CI lower**	**95 % CI upper**	**p-value**
Astro	-0.266	-0.443	-0.069	**0.009**
Endo	-0.203	-0.388	-0.002	**0.047**
Neuro-Ex	0.150	-0.052	0.340	0.146
Neuro-In	0.094	-0.109	0.289	0.363
Micro	0.174	-0.362	0.028	0.090
Oligo	0.213	0.014	0.397	**0.037**
OPC	-0.234	-0.415	-0.035	**0.022**
Per	-0.196	-0.382	0.004	0.055

Abbreviations: astrocytes (Astro), endothelial cells (Endo), microglia (Micro), excitatory neurons (Neuro-Ex), inhibitory neurons (Neuro-In), oligodendrocytes (Oligo), oligodendrocyte precursors (OPC), and pericytes (Per).

5. Supplementary References

1. Hedderich, D. M. *et al.* Sequelae of Premature Birth in Young Adults: Incidental Findings on Routine Brain MRI. *Clin Neuroradiol* **31**, 325–333 (2021).
2. Rutherford, S. *et al.* Charting brain growth and aging at high spatial precision. *Elife* **11**, (2022).
3. Bethlehem, R. A. I. *et al.* Brain charts for the human lifespan. *Nature* **604**, 525–533 (2022).
4. Frangou, S. *et al.* Cortical thickness across the lifespan: Data from 17,075 healthy individuals aged 3–90 years. *Hum Brain Mapp* **43**, 431–451 (2022).
5. Bazinet, V., Liu, Z.-Q. & Misic, B. The effect of spherical projection on spin tests for brain maps. Preprint at https://doi.org/10.1101/2024.12.15.628553 (2024).
6. Váša, F. *et al.* Adolescent Tuning of Association Cortex in Human Structural Brain Networks. *Cerebral Cortex (New York, NY)* **28**, 281–294 (2018).
7. Markello, R. D. & Misic, B. Comparing spatial null models for brain maps. *Neuroimage* **236**, 118052 (2021).
8. Alexander-Bloch, A. F. *et al.* On testing for spatial correspondence between maps of human brain structure and function. *Neuroimage* **178**, 540–551 (2018).
9. Markello, R. *et al.* *Neuromaps: Structural and Functional Interpretation of Brain Maps*. (bioRxiv, 2022). doi:10.21203/rs.3.rs-1296849/v1.
10. Burt, J. B., Helmer, M., Shinn, M., Anticevic, A. & Murray, J. D. Generative modeling of brain maps with spatial autocorrelation. *Neuroimage* **220**, 117038 (2020).
11. Fortin, J.-P. *et al.* Harmonization of cortical thickness measurements across scanners and sites. *Neuroimage* **167**, 104–120 (2018).
12. Beer, J. C. *et al.* Longitudinal ComBat: A method for harmonizing longitudinal multi-scanner imaging data. *Neuroimage* **220**, 117129 (2020).
13. Markello, R. D. *et al.* Standardizing workflows in imaging transcriptomics with the abagen toolbox. *Elife* **10**, (2021).
14. Larivière, S. *et al.* *The ENIGMA Toolbox: Cross-Disorder Integration and Multiscale Neural Contextualization of Multisite Neuroimaging Datasets*. (2020). doi:10.1101/2020.12.21.423838.
15. Virtanen, P. *et al.* SciPy 1.0: fundamental algorithms for scientific computing in Python. *Nat Methods* **17**, 261–272 (2020).
16. Seabold, S. & Perktold, J. Statsmodels: Econometric and Statistical Modeling with Python. in 92–96 (2010). doi:10.25080/Majora-92bf1922-011.
17. Vallat, R. Pingouin: statistics in Python. *J Open Source Softw* **3**, 1026 (2018).
18. Hayes, A. F. *Introduction to Mediation, Moderation, and Conditional Process Analysis*. *Methodology in the social sciences* (The Guilford Press, New York NY, 2022).

19. Hunter, J. D. Matplotlib: A 2D Graphics Environment. *Comput Sci Eng* **9**, 90–95 (2007).
20. Waskom, M. seaborn: statistical data visualization. *J Open Source Softw* **6**, 3021 (2021).
21. Larivière, S. *et al.* The ENIGMA Toolbox: multiscale neural contextualization of multisite neuroimaging datasets. *Nat Methods* **18**, 698–700 (2021).
22. Stasinopoulos, M. & Rigby, R. gamlss: Generalized Additive Models for Location Scale and Shape. *CRAN: Contributed Packages* Preprint at https://doi.org/10.32614/CRAN.package.gamlss (2012).
23. Wickham, H. *et al.* Welcome to the Tidyverse. *J Open Source Softw* **4**, 1686 (2019).
24. Rutherford, S. *et al.* The normative modeling framework for computational psychiatry. *Nat Protoc* **17**, 1711–1734 (2022).
25. Desikan, R. S. *et al.* An automated labeling system for subdividing the human cerebral cortex on MRI scans into gyral based regions of interest. *Neuroimage* **31**, 968–980 (2006).
