# Supplementary material for: Heterogeneous, temporally consistent, and plastic brain development after preterm birth
Source: Nat Commun. 2025 Sep 12;16:8269. doi: 10.1038/s41467-025-63967-1 (PMC12432136; doi:10.1038/s41467-025-63967-1)
Supplement: Supplementary file 2 — Description of Addtional Supplementary File [file 41467_2025_63967_MOESM2_ESM.pdf]

Description of Additional Supplementary File

Supplementary Data 1: Demographics Data

Demographical, clinical, and cognitive data. Data are listed for the final data sample after exclusion due to quality control. Two-sided p-values (uncorrected as well as corrected for the False Discovery Rate, FDR) are provided.

The table displays mean ± SD, except where noted. Statistical comparisons: sex with $\chi2$ statistics; SES with Mann-Whitney-U; age, GA, BW, full-scale IQ with two-sample ttests. Bold letters indicate statistical significance defined as $p < 0.05$. Abbreviations: BW, birth weight; FT, full-term; GA, gestational age; IQ, intelligence quotient; n.a., not applicable; SD, standard deviation; SES, socio-economic status; PT preterm.
