# Supplementary material for: Heterogeneous, temporally consistent, and plastic brain development after preterm birth
Source: Nat Commun. 2025 Sep 12;16:8269. doi: 10.1038/s41467-025-63967-1 (PMC12432136; doi:10.1038/s41467-025-63967-1)
Supplement: Supplementary file 4 — Reporting Summary [file 41467_2025_63967_MOESM4_ESM.pdf]

nature portfolio

Corresponding author(s): Melissa Thalhammer

Last updated by author(s): August 18, 2025

Reporting Summary

Nature Portfolio wishes to improve the reproducibility of the work that we publish. This form provides structure for consistency and transparency in reporting. For further information on Nature Portfolio policies, see our Editorial Policies and the Editorial Policy Checklist.

Statistics

For all statistical analyses, confirm that the following items are present in the figure legend, table legend, main text, or Methods section.

n/a	Confirmed	
[ ]	[x]	The exact sample size (*n*) for each experimental group/condition, given as a discrete number and unit of measurement
[ ]	[x]	A statement on whether measurements were taken from distinct samples or whether the same sample was measured repeatedly
[ ]	[x]	The statistical test(s) used AND whether they are one- or two-sided<br>*Only common tests should be described solely by name; describe more complex techniques in the Methods section.*
[ ]	[x]	A description of all covariates tested
[ ]	[x]	A description of any assumptions or corrections, such as tests of normality and adjustment for multiple comparisons
[ ]	[x]	A full description of the statistical parameters including central tendency (e.g. means) or other basic estimates (e.g. regression coefficient) AND variation (e.g. standard deviation) or associated estimates of uncertainty (e.g. confidence intervals)
[ ]	[x]	For null hypothesis testing, the test statistic (e.g. *F*, *t*, *r*) with confidence intervals, effect sizes, degrees of freedom and *P* value noted<br>*Give P values as exact values whenever suitable.*
[x]	[ ]	For Bayesian analysis, information on the choice of priors and Markov chain Monte Carlo settings
[ ]	[x]	For hierarchical and complex designs, identification of the appropriate level for tests and full reporting of outcomes
[ ]	[x]	Estimates of effect sizes (e.g. Cohen's *d*, Pearson's *r*), indicating how they were calculated

Our web collection on statistics for biologists contains articles on many of the points above.

Software and code

Policy information about availability of computer code

Data collection	No software was used in data collection
Data analysis	Data was analyzed using a combination of open source R and Python code and custom code in R, Python, and MATLAB made available on https://github.com/Melissa1909/preterm-brain-heterogeneity. With respect to all visualization and statistics represented in graphical format, unless otherwise stated, these were generated in Python v3.11.9 using "matplotlib" and "seaborn" packages. A list of all packages used can be found in Supplementary Methods S2.5. Where violin plots are used, they indicate the median and upper and lower hinges corresponding to the third and first quartiles (the 75th and 25th percentiles). The upper whisker extends from the hinge to the smallest value at most 1.5 * IQR of the hinge. Plots depicting linear associations were generated with seaborn's "regplot" function, shaded regions indicate the 95% confidence intervals of the linear relation.<br>A description of the FreeSurfer version and processing pipeline can be found in the Methods section (mainly 7.1.1. for ABCD and 7.2.3 for BLS).<br>Code from the BrainChart project (https://github.com/brainchart/Lifespan) was used to re-fit the normative models. Minor parts of code from L. Lotter were used to process data from the ABCD study (https://github.com/LeonDLotter/CTdev/blob/main/0.3_getData_ABCD.ipynb).

For manuscripts utilizing custom algorithms or software that are central to the research but not yet described in published literature, software must be made available to editors and reviewers. We strongly encourage code deposition in a community repository (e.g. GitHub). See the Nature Portfolio guidelines for submitting code & software for further information.

Data

Policy information about availability of data

All manuscripts must include a data availability statement. This statement should provide the following information, where applicable:

- Accession codes, unique identifiers, or web links for publicly available datasets
- A description of any restrictions on data availability
- For clinical datasets or third party data, please ensure that the statement adheres to our policy

Neonatal imaging data were collected for the developing Human Connectome Project (dHCP) and are available under restricted access for data privacy reasons and by regulations of the original investigators. Access can be obtained by application to the National Institute of Mental Health (NIMH) Data Archive (https://nda.nih.gov/edit_collection.html?id=3955). Further information on how to gain access can be found on the dHCP study website (https://biomedia.github.io/dHCP-release-notes/). Children imaging data were collected for the Adolescent Brain Cognitive Development (ABCD) Study and are also available under restricted access for data privacy reasons and by regulations of the original investigators. Access can be obtained by application to the NIMH Data Archive (https://nda.nih.gov/abcd/request-access). Adult imaging data were collected in-house. Raw data are protected, are not available due to data privacy laws, and cannot be publicly shared. Access to processed data can be requested on reasonable grounds; please contact the corresponding author for details. Normative reference charts for regional CTh and SA from the BrainChart project56 are publicly available on Github (https://github.com/brainchart/Lifespan). Charts for cerebral volume measures are also accessible via shinyapps (https://brainchart.shinyapps.io/brainchart/). Source Data files corresponding to each main and Supplementary Figure are provided with this paper.

Research involving human participants, their data, or biological material

Policy information about studies with human participants or human data. See also policy information about sex, gender (identity/presentation), and sexual orientation and race, ethnicity and racism.

Reporting on sex and gender	The term 'sex' was used as biological sex assigned at birth. Sex was included as covariate in all analyses. Data distributed by sex can be found in Supplementary Table S1 for all used datasets. Individual sex data will not be shared.
Reporting on race, ethnicity, or other socially relevant groupings	Socioeconomic status (SES) was used to estimate social environment during early development. For the BLS-26 dataset, SES was determined based on parental interviews around childbirth on the profession of the self-identified head of the family and the highest education held by either parent. Based on this evaluation, SES was classified into high, middle, and low. For the ABCD-10 dataset, self-reported parental education was used to estimate SES.
Population characteristics	Full population characteristics can be found in Supplementary Table S1. Age and sex were included as covariates in all analyses. Diagnosis (i.e., being born preterm or full-term) was provided individually for each dataset, procedures for obtaining these are described in the Methods section for each dataset.
Recruitment	All analyses in the present manuscript were based on existing data. Recruitment for each existing dataset is described in the Methods section.
Ethics oversight	All existing datasets already contained their own ethical oversight and therefore, no additional ethical approval was required.

Note that full information on the approval of the study protocol must also be provided in the manuscript.

Field-specific reporting

Please select the one below that is the best fit for your research. If you are not sure, read the appropriate sections before making your selection.

[x] Life sciences [ ] Behavioural & social sciences [ ] Ecological, evolutionary & environmental sciences

For a reference copy of the document with all sections, see nature.com/documents/nr-reporting-summary-flat.pdf

Life sciences study design

All studies must disclose on these points even when the disclosure is negative.

Sample size	No a-priori sample size was calculated, but we used three different datasets spanning preterm development from birth into adulthood, with two of them encompassing longitudinal assessments. By this means, we provide a sufficiently detailed view on preterm development.
Data exclusions	Exclusion criteria during data acquisition are reported in the Supplement and the Methods section for each dataset. Furthermore, during data analysis, subjects were excluded based on established quality control criteria as described in the Methods section. Missing demographic data or failure of preprocessing were other reasons for data exclusion.
Replication	Reproducibility of our findings was ensured by using two population-based pre-trained normative models as references to determine spatial heterogeneity. Several sensitivity and control analyses were conducted in three cohorts spanning cortical development.
Randomization	For the association of individual brain abnormality patterns and cellular density maps, we used spatial autocorrelation preserving null models (spin tests). For confidence interval estimations in regression plots, bootstrapping (n=10,000) was used.
Blinding	Blinding was not possible but is also not applicable to data analysis. All data analyses were conducted in a data driven manner.

Reporting for specific materials, systems and methods

We require information from authors about some types of materials, experimental systems and methods used in many studies. Here, indicate whether each material, system or method listed is relevant to your study. If you are not sure if a list item applies to your research, read the appropriate section before selecting a response.

Materials & experimental systems

n/a	Involved in the study
☒	☐ Antibodies
☒	☐ Eukaryotic cell lines
☒	☐ Palaeontology and archaeology
☒	☐ Animals and other organisms
☒	☐ Clinical data
☒	☐ Dual use research of concern
☒	☐ Plants

Methods

n/a	Involved in the study
☒	☐ ChIP-seq
☒	☐ Flow cytometry
☐	☒ MRI-based neuroimaging

Plants

Seed stocks	*Report on the source of all seed stocks or other plant material used. If applicable, state the seed stock centre and catalogue number. If plant specimens were collected from the field, describe the collection location, date and sampling procedures.*
Novel plant genotypes	*Describe the methods by which all novel plant genotypes were produced. This includes those generated by transgenic approaches, gene editing, chemical/radiation-based mutagenesis and hybridization. For transgenic lines, describe the transformation method, the number of independent lines analyzed and the generation upon which experiments were performed. For gene-edited lines, describe the editor used, the endogenous sequence targeted for editing, the targeting guide RNA sequence (if applicable) and how the editor was applied.*
Authentication	*Describe any authentication procedures for each seed stock used or novel genotype generated. Describe any experiments used to assess the effect of a mutation and, where applicable, how potential secondary effects (e.g. second site T-DNA insertions, mosiacism, off-target gene editing) were examined.*

Magnetic resonance imaging

Experimental design

Design type	Structural MRI
Design specifications	No specific experimental setup was used
Behavioral performance measures	Full-scale IQ measurements were used for the BLS-26 cohort. Data were acquired with the "Wechsler Intelligenztest für Erwachsene", the German adaptation of the Wechsler Adult Intelligence Scale, third edition. Bayley Scales of Infant and Toddler Development, Third Edition (Bayley-III) were used to estimate cognitive development of toddlers of the dHCP cohort at the age of 18 months. For children of the ABCD cohort, the NIH Toolbox Cognition Battery was used to evaluate cognitive performance at the age of 10 years. Details are described in the Methods section.

Acquisition

Imaging type(s)	Structural, T1 or T2 weighted images
Field strength	3T
Sequence & imaging parameters	Varying, description in each dataset description
Area of acquisition	whole brain

Diffusion MRI ☐ Used ☒ Not used

Preprocessing

Preprocessing software	Varying, mostly based on FreeSurfer recon-all (Infant FreeSurfer for dHCP, version 7.1.1. for ABCD, version 7.3.2. for BLS dataset)
Normalization	Varying, mostly based on FreeSurfer recon-all
Normalization template	Varying, mostly based on FreeSurfer recon-all (i.e., fsaverage)
Noise and artifact removal	Varying, mostly based on FreeSurfer recon-all

Volume censoring	None

Statistical modeling & inference

Model type and settings	Pretrained generalised additive models for location, scale, and shape (GAMLSS) were adapted to each dataset and used to assess individual deviations, incorporating age and sex as variables with study-specific random effects for term-born individuals. Furthermore, linear regression models including age and sex as covariates were implemented for group comparisons as well as associations of deviation scores and further variables such as gestational age. Deviation scores were dimensionality-reduced across 34 regions using principal component analysis to extract the main axis of variation of deviation scores across cortical regions. Spatial associations of cell-type specific gene expression maps with individual brain abnormality patterns was conducted using Spearman correlations with spatial autocorrelation-preserving p-value estimations (spin test). Furthermore, moderation analysis was used to determine the interaction effect of gestational age and socioeconomic status on the first principal component of deviation scores across cortical regions.
Effect(s) tested	Pre-trained growth trajectories of regional cortical thickness and surface area were adapted to present datasets by estimating random effects of study. For each participant and each region, individual deviation scores were derived, which served as a basis for all following analyses. Individual deviation score profiles were correlated wiith the deviation score profiles of all others using Spearman's rank correlation. Within an individual, deviation score profiles were compared longitudinally. Furthermore, individual deviation score profile derived measures were correlated with cellular density maps, measures of prematurity (gestational age, birth weight, duration of neonatal treatment index), parameters of early social environment (socio-economic status, parent-infant relationship index), and IQ within or across individuals using Spearman's rank correlation.

Specify type of analysis: ☐ Whole brain ☒ ROI-based ☐ Both

Anatomical location(s)	The surface-based Desikan-Killiany parcellation was used to determine cortical ROIs
Statistic type for inference (See Eklund et al. 2016)	Not applicable
Correction	Statistical significance was assessed using False Discovery Rate correction for multiple comparisons.

Models & analysis

n/a	Involved in the study
☒	☐ Functional and/or effective connectivity
☒	☐ Graph analysis
☐	☒ Multivariate modeling or predictive analysis

Multivariate modeling and predictive analysis	Pre-trained generalised additive models for location, scale and shape (GAMLSS) were adapted to current datasets by estimating random effects of study to estimate individual deviations in brain development after preterm birth. We used principal component analysis (PCA) to determine a main axis of variation in individual deviation scores across 34 cortical regions.
